# Treatment of periodontal biofilms via nitric oxide-augmented phototherapy

**DOI:** 10.1080/20002297.2026.2658899

**Published:** 2026-04-28

**Authors:** Courtney R. Johnson, Tsian D. Ramrattan, Casimir J. Schaefer, Rajnish Kumar, Simon J. Reinhard, Shannon M. Wallet, Mark H. Schoenfisch

**Affiliations:** a Department of Chemistry, University of North Carolina at Chapel Hill, Chapel Hill, NC, USA; b Department of Oral Biology, College of Dentistry, University of Florida, Gainesville, FL, USA; c Eshelman School of Pharmacy, University of North Carolina at Chapel Hill, Chapel Hill, NC, USA

**Keywords:** Nitric oxide, mesoporous silica nanoparticles, *S-*nitrosothiols, light-mediated release, antibacterial blue light therapy, periodontal disease, subgingival infection

## Abstract

**Objectives:**

This study investigated the antibacterial action of photo-liberated nitric oxide (NO) from primary and tertiary *S-*nitrosothiol-modified mesoporous silica nanoparticles (1° RSNO-MSNs and 3° RSNO-MSNs, respectively) against planktonic periodontal pathogens and ex vivo subgingival periodontal biofilms.

**Materials and methods:**

The antibacterial and antibiofilm efficacy of photo-liberated NO release from 1° RSNO-MSNs and 3° RSNO-MSNs was evaluated as a function of wavelength (405 and 455 nm), irradiance (400–1000 mW cm^−2^), and time (0–15 min) against *Aggregatibacter actinomycetemcomitans, Porphyromonas gingivalis,* and ex vivo subgingival biofilms. Time course, biofilm dispersion, and biofilm eradication assays were employed to evaluate the synergistic impact of antibacterial blue light therapy (aBLT) and photo-liberated NO.

**Results:**

The addition of photo-initiated NO release with aBLT significantly improved antibiofilm activity, with irradiation of 405 nm at 1000 mW cm^−2^ demonstrating the most efficacious NO liberation from both 1° RSNO-MSNs and 3° RSNO-MSNs. While aBLT demonstrated antibacterial activity alone, the addition of NO was crucial for biofilm dispersal and eradication.

**Conclusions:**

The in vitro and ex vivo results demonstrate, for the first time, the utility of a dual-action aBLT and NO-releasing system for treating periodontal pathogens and biofilms.

## Key messages

- Photo-initiated nitric oxide (NO) release from RSNO-modified mesoporous silica nanoparticles enables wavelength- and irradiance-dependent control over NO-release kinetics and payloads, allowing precise modulation of antibacterial activity.
- Antibacterial efficacy against dental biofilms is governed not only by total NO payload but by NO-release kinetics, with sustained NO release enabling superior biofilm penetration and eradication compared to rapid, high-flux release at equivalent NO doses.
- Combining antimicrobial blue light therapy with photo-initiated NO delivery overcomes intrinsic biofilm protective mechanisms, enabling effective eradication of resilient periodontal biofilms under clinically relevant irradiation conditions.

## Graphical abstract

**Figure:**
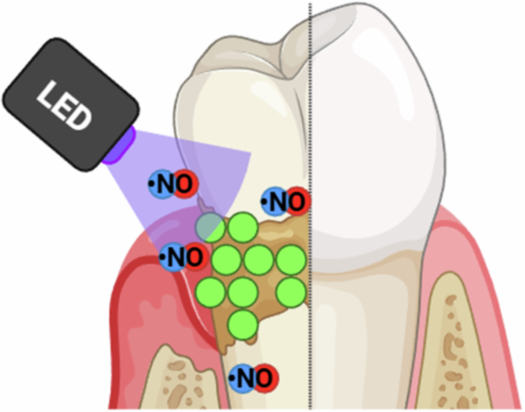

## Introduction

Periodontal disease is a chronic inflammatory disease of the oral mucosa affecting over 1.1 billion people worldwide with a substantial health care burden [1]. Initiated by subgingival microbiome dysbiosis and the formation of dental plaque biofilms, periodontal disease occurs over distinct stages [2,3]. When left untreated, periodontal disease progresses from gingivitis to moderate and advanced periodontitis, characterized by infection beyond the gingiva in the periodontal ligament, cementum, or alveolar bone [4,5]. Ongoing tissue inflammation and damage lead to progressive bone loss, tooth migration and loss, chronic inflammation, and increased occurrence of other noncommunicable diseases, including cardiovascular diseases, rheumatoid arthritis, diabetes mellitus, and respiratory diseases [1,6].

Biofilm formation is a process wherein bacteria first adhere to the tooth, irreversibly attach, and then produce an exopolysaccharide (EPS) matrix that promotes further colonization and propagation [7,8]. The formation of the EPS matrix results in a barrier or blockade through which traditional therapeutics struggle to penetrate [9]. Additionally, the microenvironment of the biofilm alters physiochemical conditions, impacts antibiotic tolerance, and encourages quorum sensing across species, whereby pathogenic species are able to flourish [8,10]. Through quorum sensing and interspecies interaction, pathogenic species proliferation is encouraged, thereby increasing virulence and inducing periodontal inflammation [11–13]. In turn, pathogenic proliferation increases bacterial load, resistance to host defences and limits therapeutic intervention [11].

Effective treatment of dental biofilms remains a challenge because these highly organized microbial communities develop multiple protective mechanisms that confer resistance to conventional therapies, necessitating greater intervention than unattached planktonic bacteria [14]. While gingivitis is reversible and may be effectively treated through routine dental cleanings and consistent daily oral hygiene practices (i.e. brushing, flossing), periodontitis is a chronic, irreversible disease that requires ongoing management to prevent further tissue destruction and bone loss [2]. Current therapeutic approaches include invasive procedures such as root scaling and root planing combined with adjuvant therapies such as locally delivered antimicrobials and antibiotics, systemic antibiotics, or systemic host-modulating agents [2,4,5]. While the use of antimicrobials (e.g. chlorhexidine) and antibiotics (e.g. minocycline, doxycycline) has improved therapeutic outcomes for individuals with periodontitis, antibiotic resistance and poor biofilm penetration limit their long-term efficacy and prevent complete eradication, thereby risking recurrent infection [15].

Antibacterial blue light therapy (aBLT) has been previously investigated to overcome the limitations of conventional treatments. Natively, bacteria are susceptible to wavelengths of light in the blue region (400–470 nm) because of the formation of reactive oxygen species (ROS) upon photo-exposure [16]. As highly reactive small molecules, ROS (i.e. ∙OH, ^1^O_2_, O_2_^-^ and H_2_O_2_) are generated when endogenous photosensitizers, porphyrins, are excited to a triplet state and provide bactericidal activity against both antibiotic-resistant and -susceptible strains [17]. However, aBLT efficacy is directly related to endogenous porphyrin abundance, which is dependent on bacterial species [18]. Species-specific optimization of treatment parameters is thus required to achieve therapeutic utility [18]. Periodontal pathogens have previously been shown to be susceptible to aBLT, but these studies largely focused on bacteria in the planktonic state [19–22]. Compared with biofilms, aBLT exhibits reduced efficacy due to limited light penetration, decreased diffusion of ROS through the matrix, and species-dependent variations in the levels of endogenous photosensitizers [23,24]. To this end, aBLT is best utilized with complementary antimicrobial or photo-chemical strategies to achieve effective disruption of mature biofilms and ensure therapeutic efficacy.

Photo-sensitive nitric oxide (NO) donors offer the opportunity to harness the therapeutic advantages of aBLT while delivering NO in an on-demand fashion [25]. As a potent antimicrobial agent that is natively produced within the body, NO elicits bactericidal action through both oxidative and nitrosative mechanisms to promote DNA damage, protein nitrosation, and lipid peroxidation [26–28]. Owing to NO’s multiple mechanisms of antibacterial action, the development of traditional antimicrobial resistance is unlikely and has not been reported to date [29,30]. The NO-release kinetics of photo-sensitive *S-*nitrosothiols (RSNOs) are dependent on the donor structure and specific environmental conditions such as temperature and light exposure [25]. The unique photo-labile nature of RSNOs has the potential for spatial and temporal control of NO production when an external stimulus, such as light, is employed [31,32]. Homolytic cleavage of the S–N bond occurs, breaking down the NO donor to NO and a thiyl radical [32–34]. Tertiary RSNOs (3° RSNOs) are generally more stable than their primary RSNO (1° RSNO) counterparts, largely as a result of increased steric interactions around the thiol group [33–35]. The photo-liberation of NO is thus tunable beyond light parameters (i.e. wavelength, irradiance, photo-exposure time) alone by increasing the steric hinderance from 1° RSNO to 3° RSNO, and thus the stability of the NO donor, to expand the library of distinct NO release profiles and kinetics. The ability to control both the rate and payload of NO delivery may enhance NO penetration into the dense extracellular matrix, minimizing potential cytotoxic effects on host tissues. *S-*nitrosothiols therefore function as photo-responsive NO prodrugs capable of site-specific activation to achieve antibacterial NO concentrations (µM) [36].

Combining the effects of aBLT and photo-sensitive RSNO donors offers a synergistic approach to overcome the current challenges presented by dental biofilms. To improve localized delivery, mesoporous silica nanoparticles (MSNs) may serve as versatile carriers that enable surface functionalization of 1° RSNO and 3° RSNOs, provide high NO payloads, and facilitate controlled NO release [37,38]. Previously, we demonstrated the synergistic antibacterial effects of aBLT and photo-initiated NO release from 1° RSNO-MSNs against planktonic aerobic pathogens [39]. Herein, we aimed to evaluate the influence of light parameters and RSNO-functionalized MSNs to develop a suite of NO-release kinetics and payloads. The therapeutic potential of photo-initiated NO release for periodontal disease was determined via antibacterial and cytocompatibility studies, including the use of ex vivo periodontal biofilms, to investigate dispersion and eradication.

## Materials and methods

### Synthesis of NO-releasing mesoporous silica nanoparticles

The synthesis and characterization of 1° RSNO- and 3° RSNO-modified MSNs have been previously described [37,39]. Briefly, unmodified MSNs were synthesized using a modified Stöber method in which tetraethylorthosilicate (TEOS; Gelest, Morrisville, PA) condenses around a liquid crystal template formed by cetyltrimethylammonium bromide (CTAB; Sigma–Aldrich, St. Louis, MO) and ammonium hydroxide (NH_4_OH; Fisher Scientific, Waltham, MA) in ethanol (EtOH; Fisher Scientific) and water (17.2:51.7:1 molar ratio EtOH:H_2_O:NH_4_OH). The particles were washed with EtOH, and the CTAB was extracted via ion exchange with acidified EtOH. The resulting MSNs were then dried in vacuo overnight. To functionalize free primary thiols onto the surface of the MSNs (1° SH-MSNs), 3-mercaptopropyltrimethoxysilane (MPTMS; Gelest) was surface grafted onto the MSNs in dimethylformamide (DMF; Fischer Scientific) by refluxing for 16 h at 160 °C. The functionalized particles were washed with EtOH and dried in vacuo overnight. To modify MSNs with free tertiary thiols (3° SH-MSNs), *N*-acetyl-D-penicillamine thiolactone (NAP-thiolactone) was first synthesized following previously described methods [32]. Briefly, D-penicillamine and anhydrous pyridine were stirred together for 30 min before the addition of acetic anhydride (Sigma‒Aldrich). After stirring overnight, the solvents were removed via rotary evaporation. The crude product was then redissolved in chloroform (Fisher Scientific) and washed with hydrochloric acid (HCl; Fisher Scientific). The chloroform layer was dried over anhydrous sodium sulphate (Fisher Scientific) before rotary evaporation. The product was then purified by crystallization in EtOH and hexane (Fisher Scientific). The resultant NAP-thiolactone was dissolved in anhydrous dichloromethane (DCM; Fisher Scientific), and an equimolar amount of (3-aminopropyl)trimethoxysilane (APTMS, Sigma–Aldrich) was added dropwise to form 2-acetamido-3-mercapto-3-methyl-*N*-(3-(trimethoxysilyl)propyl)-butanamide (NAPTMS). After stirring for 4 h at room temperature, the DCM was removed using a rotary evaporator. The pure NAPTMS was surface grafted onto the MSNs in toluene by refluxing for 16 h at 120 °C. The particles were washed with EtOH as previously described and dried in vacuo [37].

The functionalization of NO onto the 1° SH-MSNs and 3° SH-MSNs occurred through a nitrosation reaction. Thiol-containing MSNs were suspended in methanol (MeOH; Fisher Scientific) and HCl. Sodium nitrite (NaNO_2_; Sigma–Aldrich) in water was added dropwise. Ethylenediaminetetraacetic acid (EDTA; Sigma–Aldrich) served as a metal chelator to prevent premature NO release. The reaction was stirred on ice for 1 h, and the product was then washed with water and EtOH. The 1° RSNO-MSNs and 3° RSNO-MSNs were dried under vacuum for 1 h in the dark to minimize premature NO donor breakdown. The dried RSNO-MSNs were stored at -80 °C until further use.

### Photolytic NO release

A Sievers 280i chemiluminescent nitric oxide analyzer (NOA; Frederick, CO) was used to measure NO release from the RSNO-MSNs in real time. In phosphate-buffered saline (PBS, 10  mM, pH 7.4), 1° RSNO-MSNs or 3° RSNO-MSNs were added to the reaction flask, which was placed in a custom 3D printed holder directly above an LED source (5 mm). The holder was allowed for consistent illumination through a 12.5 mm hole to avoid unwanted photo-irradiation. Light-emitting diodes with nominal wavelengths of 405 and 455 nm were purchased from ThorLabs, Inc. (Newton, NJ). Photo-initiated NO-release experiments were conducted using separate LED sources with thermal heat sinks powered by an LED-driver. A Si photodiode detector (PM16-121; ThorLabs, Inc.) was used to confirm LED irradiance prior to experimentation. The samples were irradiated for 15  min with 405 or 455  nm at 400, 800, or 1000 mW cm^−2^, with NO release measured in real-time.

### Quantum yield of NO release

Equation (1) was employed to determine the quantum yield of NO release from 1° RSNO-MSNs or 3° RSNO-MSNs upon photo-exposure at 405 or 455 nm at 400, 800, or 1000 mW cm^−2^, where the number of NO molecules released was represented as *n*_NO_ and the number of incident photons as *n*_*P*_ *. h* is Planck’s constant (6.626 × 10^−34^ J ∙ s), *c* is the speed of light (299792458 m ∙ s^−1^), *I* is the irradiance (W ∙ m^−2^), and *t* is the irradiation time (s) [40,41]. The irradiation area (m^2^) is denoted as *A*, and the wavelength of light (m) is λ:

(1) $${\mathrm{QY}}_{\mathrm{NO}}=\frac{n_{\mathrm{NO}}}{n_{P}}=\;\frac{n_{\mathrm{NO}}\times h\times c}{I\times t\times A\times \lambda }$$

### Temperature change upon LED exposure

The change in solution temperature following photo-exposure was evaluated at intervals of 1–15 min using a digital thermometer (Dual Channel K-Type Thermocouple Thermometer; Amazon, Seattle, WA) in solution volumes of 1 mL (i.e. photo-based experiment volumes in vitro).

### In vitro photo-toxicity

Human oral epithelial cells (HOEC) were cultured in HOEC culture complete media with serum and antibiotics (Celprogen; Torrance, CA). Human periodontal ligament fibroblasts (HPLF) were cultured in fibroblast media with growth supplements (1% v/v FBS, 1% v/v PSA, and FGS) (Sciencell; Carlsbad, CA). The cells were incubated in 5% v/v CO_2_ under humidified conditions at 37 °C. Cell suspensions of 2.5 × 10^5^ and 2 × 10^5^ cells mL^−1^ were prepared for HOECs and HPLFs, respectively, with 1 mL aliquots added to 24-well tissue culture-treated polystyrene plates and incubated to allow the cells to adhere. Once adhered, the cells were treated with 2 mg mL^−1^ MSNs and exposed to LED sources for 15  min at 400, 800, or 1000 mW cm^−2^. The cells were incubated for 24 h immediately following irradiation, with viability determined using resazurin sodium salt solution (20 µL of 1 mg mL^−1^ per well). Fluorescence was measured at 544 nm excitation and 590 nm emission wavelengths using a Molecular Devices SpectraMax M2 spectrophotometer (San Jose, CA), and cell viability was determined using Equation (2):

(2) $$\%\;\mathrm{cell viability}=\frac{{\mathrm{Fluorescence}}_{\mathrm{sample}}-{\mathrm{Fluorescence}}_{\mathrm{blank}}}{{\mathrm{Fluorescence}}_{\mathrm{control}}-{\mathrm{Fluorescence}}_{\mathrm{blank}}}\times 100$$

### In vitro time-based planktonic eradication assay

Bacterial cultures of *A. actinomycetemcomitans* (ATCC 43717; American Type Culture Collection; Manassas, VA) and *P. gingivalis* (ATCC 33277; American Type Culture Collection) were grown from frozen stocks (-80 °C) on Difco Brain Heart Infusion agar (BHI; Avantor Sciences, Radnor, PA) or tryptic soy agar (TSA; Avantor Sciences) supplemented with 5% v/v defibrinated sheep blood (Avantor Sciences), 5 mg L^−1^ hemin (Sigma–Aldrich) and 0.5 mg L^−1^ vitamin K3 (Sigma–Aldrich), respectively. Isolated colonies were sampled and grown in Difco BHI broth (Avantor Sciences) or Wilkins Chalgren broth (WCB; Sigma–Aldrich), respectively, for 48–72 h to achieve log phase (OD_600_ 0.2–0.8). Bacteria were then diluted to 5 × 10^7^ CFU mL^−1^ in 10% v/v broth before transferring 1 mL to 24-well polystyrene plates prior to experimentation. Wells containing only bacteria, 1° SH-MSNs, 3° SH-MSNs, 1° RSNO-MSNs, or 3° RSNO-MSNs (2 mg mL^−1^) were irradiated with 405 or 455 nm light at 400, 800, or 1000 mW cm^−2^ for 15 min or maintained in the dark. Aliquots (15 µL) were removed from the well at 1, 3, 5, 10, and 15 min and serially diluted 10–100,000 times. The dilutions were plated on agar and incubated under anaerobic conditions at 37 °C for 48–72 h prior to colony enumeration for pathogen viability.

### Development of ex vivo biofilm

Subgingival plaque samples were collected from periodontitis patients at the University of Florida College of Dentistry. Patient recruitment and sample collection were approved by the institutional review board. Periodontally diseased participants will be diagnosed with moderate to severe periodontitis, which is defined by: 4 mm probing depth, 2 mm and clinical attachment level, 2 mm, radiographic bone loss, tooth loss due to periodontitis ≤ 4 teeth, and at least 30% of sites with bleeding-on-probing (BOP). The plaque sample was mixed with glycerol to yield an inoculum from which biofilms could be grown. The inoculum was stored at -80 °C prior to use. A sample of the biofilm inoculum was grown under anaerobic conditions in BHI broth for 24 h to an OD_600_ of 0.5 prior to performing a 1:100 dilution. A 1 mL aliquot of this dilution was added to tissue culture-treated 24-well plates and incubated for 24 h to facilitate biofilm formation.

### Ex vivo biofilm dispersion

Biofilm mass was determined via crystal violet staining to investigate the impact of photo-initiated NO release on biofilms. Corning Transwell inserts (Fischer Scientific) were positioned directly above the developed ex vivo biofilm surface. For the Transwell, 100 µL of BHI was used to deliver 2 or 8 mg of MSNs (i.e. 1° SH-MSNs, 3° SH-MSNs, 1° RSNO-MSNs, or 3° RSNO-MSNs). The samples were irradiated at 405 nm at 1000 mW cm^−2^ for 15 min and incubated under anaerobic conditions for 24 h. The supernatant was removed from the biofilm, washed 1x with PBS to remove planktonic bacteria, and heat fixed for 1 h at 70 °C. Biofilms were stained with 0.1% v/v crystal violet (Sigma–Aldrich) for 15 min, washed with sterile water to remove residual crystal violet, and then, the crystal violet was eluted with 30% v/v acetic acid (Fisher Scientific). The absorbance was measured at 575 nm to quantify the remaining biofilm following treatment.

### Ex vivo biofilm viability

Biofilms were also grown as described above to determine the metabolic activity of biofilms post-treatment with NO-releasing materials and 405 nm light. Directly to the well, 2 or 8 mg mL^−1^ (1 mL) of MSNs were added, and the mixture was exposed to 405 nm light at 1000 mW cm^−2^ for 15 min. After 24 h incubation post-treatment, Transwell inserts were removed, and resazurin solution (20 µL of 1 mg mL^−1^) was added to each well. The samples were incubated anaerobically for 1 h, and fluorescence was measured, and viability was calculated as previously described.

### Ex vivo biofilm imaging

Ex vivo biofilm samples were grown on sterile paper disks (Sigma–Aldrich) for imaging studies. Briefly, 1 mL of diluted biofilm inoculum was added to each well containing sterile disks and incubated for 24 h. Nitric oxide-releasing MSNs (i.e. 1° RSNO-MSNs and 3° RSNO-MSNs) were added to Transwell inserts, and the samples were irradiated with 405 nm at 1000 mW cm^−2^ for 15 min and then incubated for 24 h under anaerobic conditions prior to fixing. After 24 h post-treatment, the Transwell inserts were removed, and the biofilm-containing disks were washed with sterile water and fixed in preparation for scanning electron microscopy (SEM) imaging based on a previous protocol [42]. The samples were fixed overnight in 4% w/v glutaraldehyde (Sigma–Aldrich), 2% w/v paraformaldehyde (Sigma–Aldrich), 0.15% w/v alcian blue (Sigma–Aldrich) and 1% w/v tannic acid (Sigma–Aldrich) in 0.1 M sodium cacodylate buffer (pH 7.4; Sigma–Aldrich). The samples were then dehydrated gradually in 10 min increments in 30, 50, 70, 90, 95, and 100% v/v EtOH. Finally, the samples were dried in 50:50 EtOH:hexamethyldisilazane (Sigma–Aldrich) for 10 min, 100% hexamethyldisilazane for 10 min, and dried for 3 d. The samples were sputter-coated with 15 nm gold/palladium before imaging with a S-4700 cold cathode field-emission scanning electron microscope.

### Ex vivo biofilm qPCR

gDNA was isolated using a DNeasy 96 PowerSoil Pro Kit, according to the manufacturers’ instructions. The gDNA was then probed for consensus 16S rRNA using Cyber Green and the following primers For: AGA GTT TGA TCC TGG CTC AG Rev: ACG GCT ACC TTG TTA CGA CTT and thermocycle conditions of 95 °C for 4 min followed by 35 cycles of 96 °C for 1 min; 60 °C for 45 s; and 72 °C for 2 min. The relative abundance was calculated by subtracting the CT value from the maximum number of cycles (i.e. 35).

### Evaluation of synergistic interactions using bliss independence

To determine the synergistic, antagonistic or independent behaviour of the treatment, the predicted treatment efficiency (Y_LED/NO,P_) was calculated according to the Bliss independence model [43] (Equation 3), where Y_LED_ is the LED treatment and Y_NO_ is the NO-releasing material.

(3) $$Y_{\mathrm{LED}/\mathrm{NO},P}=Y_{\mathrm{LED}}+Y_{\mathrm{NO}}-Y_{\mathrm{LED}}Y_{\mathrm{NO}}$$

The observed combined antibacterial activity (Y_LED/NO,O_) may then be compared to the Bliss-predicted activity (Equation 4). The synergistic method is defined as ΔY_LED/NO_ > 0, the antagonistic method is defined as ΔY_LED/NO_ < 0, and ΔY_LED/NO_ = 0 is independent.

(4) $${\Delta Y}_{\mathrm{LED}/\mathrm{NO}}=Y_{\mathrm{LED}/\mathrm{NO},O}-Y_{\mathrm{LED}/\mathrm{NO},P}$$

### Porphyrin extraction for fluorescence and absorbance measurements

Porphyrin extraction was carried out following previously established protocols [44,45]. Briefly, *A. actinomycetemcomitans*, *P. gingivalis*, and the subgingival ex vivo samples (150 mL) were grown anaerobically for 48–72 h to an OD_600_ of ≥ 0.7. The cells were centrifuged (7000 g) at 4 °C, washed 2x with chilled Tris buffer (0.05 M Tris pH 8.2) with 2 mM EDTA, and resuspended in 1 mL of ethyl acetate/acetic acid (3:1, v/v). The bacterial cells were then lysed by sonication and chilled on ice. The cell debris was removed via centrifugation and washed with 1 mL of water, and the aqueous bottom layer was discarded. The remaining porphyrins were solubilized in 100 µL of 3 M HCl. The absorbance was measured between 350–700 nm, and the fluorescence was detected with 405 nm excitation and 400–700 nm emission. Porphyrin levels were normalized to the initial OD_600_.

### Statistical analysis

The data are reported as the mean ± standard deviation from *n* ≥ 3 syntheses or the mean ± standard error of the mean from *n* ≥ 3 biological replicates for *in vitro* and ex vivo studies unless otherwise noted. Significance testing was performed using a one-way ANOVA in GraphPad Prism 10 (San Diego, CA). Non-linear regression (normalized response with variable slope) analysis was performed to determine the IC50 values. Significance testing for photothermal effects in planktonic bacterial cultures and cell experiments was performed using Student’s t-test. Significance testing for phototoxicity cell experiments and ex vivo biofilm viability experiments was performed using an ordinary one-way ANOVA with Dunnet’s correction for multiple comparisons. Significance testing for bacterial abundance was performed using an ordinary one-way ANOVA with Šídák’s correction for multiple comparisons.

Significance levels for statistical methods are denoted: *p < 0.05, **p < 0.01, ***p < 0.005.

## Results

### Photo-driven NO release

**Figure 1.**
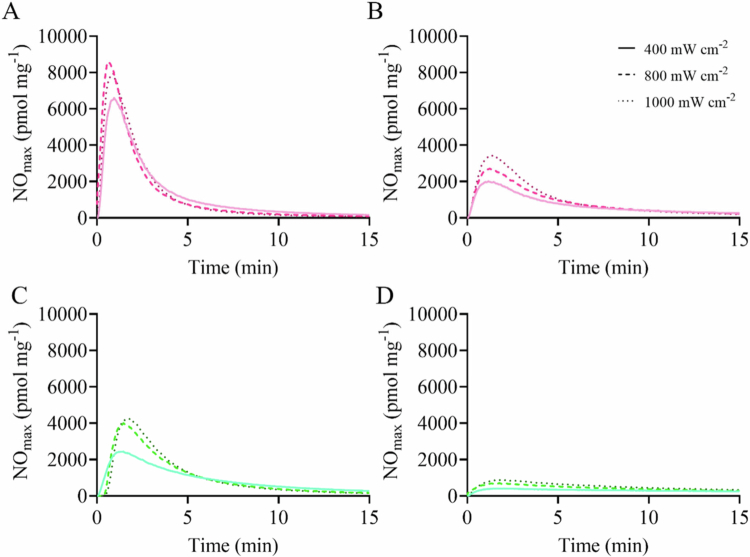
Real-time NO-release profiles of (A, B) 1° RSNO-MSNs and (C, D) 3° RSNO-MSNs. The samples were irradiated for 15  min at (A, C) 405 nm or (B, D) 455 nm. Irradiances of 400 mW cm^−2^ (solid line), 800 mW cm^−2^ (dashed line), and 1000 mW cm^−2^ (dotted line) were investigated. Nitric oxide-release measurements were conducted at pH 7.4 in 10 mM PBS at 37 °C.

Two RSNO-modified MSNs of different stabilities were selected for study (i.e. 1° RSNO-MSNs and 3° RSNO-MSNs) to determine the therapeutic potential of photo-initiated NO release, as these systems result in distinct NO-release kinetics based on NO donor stability 35. Particles were synthesized and characterized as previously reported via SEM, CHNS elemental analysis, and NO analysis (Figure S1 and Tables S1, S2) [37,39]. Two aBLT wavelengths, 405 and 455 nm, and three irradiances (i.e. 400, 800, and 1000 mW cm^−2^) were investigated to enable a systematic evaluation of photo-initiated NO release. The selected wavelengths correspond to regions of strong absorption by endogenous bacterial porphyrins, enabling ROS generation for aBLT, while simultaneously providing sufficient photon energy to induce homolytic cleavage of the S–N bond within the RSNO moiety, resulting in photo-initiated NO release [46,47]. The irradiances selected were used to evaluate both the influence of light intensity on NO-release kinetics and the rate of RSNO photolysis. In both 1° RSNO-MSN and 3° RSNO-MSN samples, NO-release kinetics and NO payloads were influenced by photo-exposure parameters. The shorter aBLT wavelength of 405 nm demonstrated rapid NO-release kinetics compared to 455 nm, with [NO]_max_ increasing 2–3x for 405 nm over 455 nm in the 1° RSNO-MSNs (Figure 1A, 1B; Table S3). The total amount of NO liberated by photo-exposure (i.e. [NO]_LED_) was greater for 405 nm than 455 nm (Table 1). Similarly, [NO]_max_ of 3° RSNO-MSNs increased approximately 5x when irradiated with 405 nm light over 455 nm (Figure 1C, 1D; Table S3). The irradiance (between 400 and 1000 mW cm^−2^) also resulted in diverse NO-release kinetics and payloads. Indeed, increasing the irradiance resulted in more elevated [NO]_max_ and [NO]_LED_ values (Figure 1; Table 1 and 2), regardless of the RSNO-MSN type and light frequency. The greatest irradiance resulted in the largest [NO]_LED_, with its effects most apparent at the 455 nm wavelength (Table 1, Table 2). Conversely, the lowest irradiance (i.e. 400 mW cm^−2^) resulted in diminished [NO]_max_ and [NO]_LED_ for all RSNO-MSN samples investigated. The stability of the RSNO-MSN system also altered the NO release during photo-exposure. Primary RSNO-MSNs rapidly liberated NO compared to their 3° RSNO-MSN counterparts at comparable irradiances and wavelengths (Figure 1). The instantaneous [NO]_max_ was 1–2x greater for 1° RSNO-MSNs than 3° RSNO-MSNs when irradiated with 405 nm light and 3–5x greater for 455 nm exposure.

**Table 1.** On-demand NO release of 1° RSNO-MSNs.^a^

		400 mW cm^−2^	800 mW cm^−2^	1000 mW cm^−2^
**Wavelength (nm)**	**Time** **(min)**	**NO**_**LED**_ **(μmol mg**^**−1**^**)**^b^	**NO**_**LED**_ **(μmol mg**^**−1**^**)**^b^	**NO**_**LED**_ **(μmol mg**^**−1**^**)**^b^
405	1	0.25 ± 0.06	0.39 ± 0.05	0.32 ± 0.001
	3	0.76 ± 0.08	0.89 ± 0.03	0.88 ± 0.02
	5	0.94 ± 0.07	1.03 ± 0.03	1.03 ± 0.02
	10	1.12 ± 0.07	1.15 ± 0.03	1.14 ± 0.04
	15	1.19 ± 0.07	1.19 ± 0.04	1.17 ± 0.05
455	1	0.07 ± 0.02	0.09 ± 0.02	0.10 ± 0.01
	3	0.28 ± 0.03	0.37 ± 0.09	0.46 ± 0.01
	5	0.40 ± 0.04	0.52 ± 0.10	0.64 ± 0.02
	10	0.56 ± 0.06	0.71 ± 0.12	0.82 ± 0.03
	15	0.66 ± 0.06	0.80 ± 0.11	0.91 ± 0.03

^a^ Error represents the standard deviation for *n* ≥ 3 analyses.

^b^ Total NO released upon photo-irradiation.

**Table 2.** On-demand NO release of 3° RSNO-MSNs.^a^

		400 mW cm^−2^	800 mW cm^−2^	1000 mW cm^−2^
**Wavelength (nm)**	**Time** **(min)**	**NO**_**LED**_ **(μmol mg**^**−1**^**)**^b^	**NO**_**LED**_ **(μmol mg**^**−1**^**)**^b^	**NO**_**LED**_ **(μmol mg**^**−1**^**)**^b^
405	1	0.07 ± 0.04	0.06 ± 0.02	0.04 ± 0.01
	3	0.33 ± 0.06	0.47 ± 0.07	0.48 ± 0.04
	5	0.50 ± 0.07	0.68 ± 0.07	0.72 ± 0.06
	10	0.73 ± 0.08	0.90 ± 0.05	0.92 ± 0.08
	15	0.85 ± 0.07	0.97 ± 0.05	0.99 ± 0.09
455	1	0.01 ± 0.001	0.03 ± 0.01	0.03 ± 0.01
	3	0.06 ± 0.003	0.11 ± 0.01	0.12 ± 0.02
	5	0.10 ± 0.01	0.17 ± 0.01	0.21 ± 0.03
	10	0.20 ± 0.01	0.30 ± 0.01	0.37 ± 0.05
	15	0.28 ± 0.02	0.40 ± 0.01	0.48 ± 0.06

^a^ Error represents the standard deviation for *n* ≥ 3 analyses.

^b^ Total NO released upon photo-irradiation.

Photolytic efficiency was evaluated for both wavelengths at all irradiances (Table S4). As the irradiance increased, the efficiency of photolytic RSNO decomposition decreased. This phenomenon was observed in the 405 nm irradiated 1° RSNO-MSN samples, where no statistical difference was observed in [NO]_LED_ across each irradiance at any given time point (Table 1). Owing to the release efficiency afforded by 405 nm irradiation at 400 mW cm^−2^, increasing the available photons (i.e. more photons as irradiance increases) did not impact NO-release kinetics or NO payloads. However, the LED wavelength greatly altered the photolytic efficiency. For example, 405 nm light more effectively liberated NO from the RSNO-MSNs than 455 nm and subsequently improved the NO quantum yield.

### Nanoparticle concentration screen

The cytocompatibility of native (i.e. without light stimulation) NO release from 1° RSNO-MSNs and 3° RSNO-MSNs was evaluated against HOEC and HPLF cell lines (Figure S3). In a monolayer culture, the half-maximal inhibitory concentrations (i.e. concentration at which 50% of the cells are alive; IC_50_) were 0.23 ± 0.01 and 0.92 ± 0.22  mg mL^−1^ against HOECs for 1° RSNO-MSNs and 3 ° RSNO-MSNs, respectively. Against HPLFs, the IC_50_ values were found to be 0.27 ± 0.02 and > 16 mg mL^−1^ for 1° RSNO-MSNs and 3° RSNO-MSNs, respectively (Figure S3).

The antibacterial activity of 1° RSNO-MSNs and 3° RSNO-MSNs was screened against *A. actinomycetemcomitans* and *P. gingivalis* using a minimum bactericidal concentration assay (MBC), defined as a 3-log (i.e. 99.9%) reduction in pathogen viability (Table S5). Against *A. actinomycetemcomitans*, 4 mg mL^−1^ of 1° RSNO-MSNs were required to achieve a 3-log reduction. No antibacterial activity was observed for 3° RSNO-MSNs. When challenged against *P. gingivalis,* 2 and 8 mg mL^−1^ were needed to eradicate the pathogen using 1° RSNO-MSN and 3° RSNO-MSNs, respectively. To balance therapeutic efficacy (i.e. potential antibacterial activity) and cytocompatibility, 2 mg mL^−1^ was selected for in vitro photo-eradication studies.

### Photothermal impact

While LEDs convert a significant portion of electrical energy into light, a large fraction is converted into heat, necessitating heat sinks or a thermally conductive material to pull heat away from the LED [48]. Despite the incorporated heat sink, photothermal effects may occur when high irradiances are exposed to the solution for an extended period (i.e. 15 min). As the irradiance increased, a greater photothermal effect was observed, with 1000 mW cm^−2^ irradiance increasing the solution temperature to more than 2x compared to 400 mW cm^−2^ for both wavelengths (Figure S4). A wavelength-dependence was also noted, wherein 405 nm resulted in greater temperature differences than 455 nm at comparable times and irradiances. Irradiation with 405 nm at 1000 mW cm^−2^ had the most profound effect by increasing solution temperature ~10 °C.

To verify that photothermal effects do not impact antibacterial activity, planktonic *P. gingivalis* and *A. actinomycetemcomitans* were incubated at 47 °C (i.e. 10 °C change in temperature) for 15 min. No difference in viability was observed (Figure S5). Given their increased sensitivity compared to HPLFs, HOECs were evaluated to confirm that photothermal treatment did not induce cytotoxic effects (Figure S6). No significant difference in cell viability was observed. As such, photothermal effects do not contribute to antibacterial activity nor cytotoxic effects.

### Cytocompatibility of photo-initiated NO release

**Figure 2.**
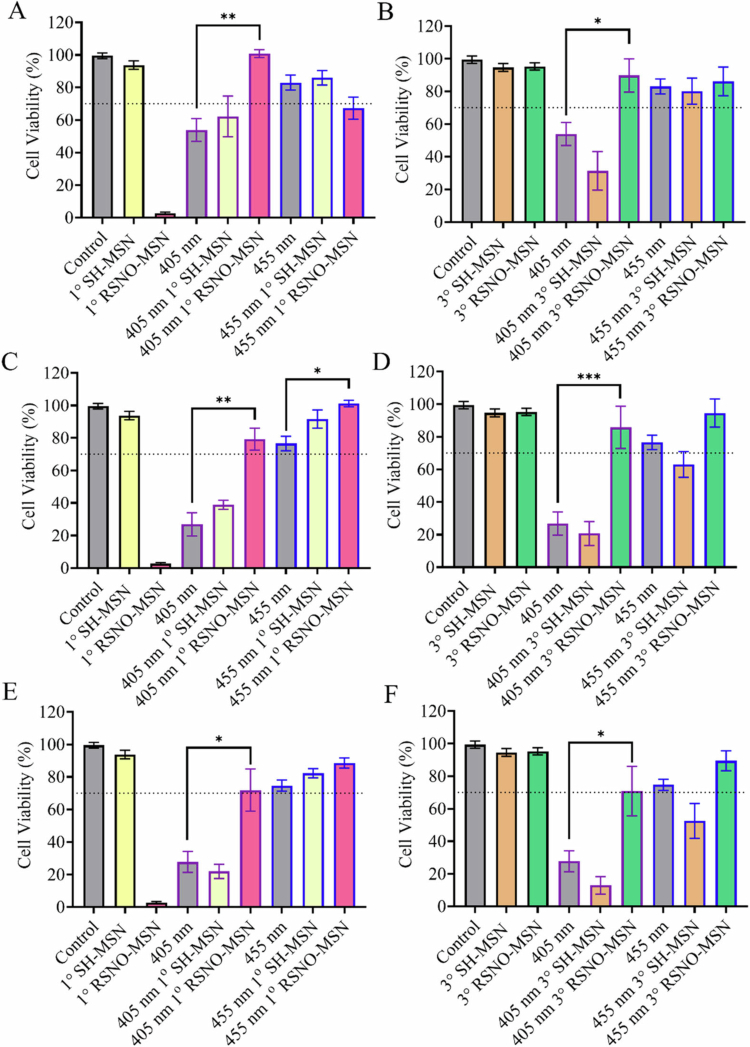
Photo-toxicity studies of 2 mg mL^−1^ 1° RSNO-MSNs and 3° RSNO-MSNs were performed 24 h post treatment against HPLFs cell lines. The cells were irradiated with (A, C, E) 405 nm or (B, D, F) 455 nm light with irradiances of (A, B) 400 mW cm^−2^, (C, D) 800 mW cm^−2^, or (E, F) 1000 mW cm^−2^. The samples contained no MSNs (grey), 1° SH-MSNs (yellow), 1° RSNO-MSNs (pink), 3° SH-MSNs (orange), or 3° RSNO-MSNs (green). The dashed line indicates 70% viability (i.e. cytocompatible). Error bars represent the standard error of the mean for *n* ≥ 3 biological replicates.

The phototoxicity of aBLT and NO release was evaluated at 405 and 455 nm for 400, 800, and 1000 mW cm^−2^ irradiances against HPLFs and HOECs. Independent of photo-exposure, the SH-MSNs of both species were well tolerated by HPLFs (Figure 2). While viability remained high for 3° RSNO-MSN, 1° RSNO-MSNs demonstrated cytotoxic behaviour (Figure 2). Photo-exposure of aBLT independently was well tolerated at 455 nm for all irradiances (Figure 2B, 2D, 2F), but 405 nm proved photo-toxic (Figure 2A, 2C, 2E). Upon the addition of RSNO-MSNs with aBLT, HPLF viability improved compared to photo-exposure alone (Figure 2), with the greatest effect seen in the 405 nm studies, where 70% viability (i.e. cytocompatible) was achieved.

Against HOECs, non-NO-releasing 1° SH-MSNs and 3° SH-MSNs were well tolerated. However, NO release from the 1° RSNO-MSNs resulted in decreased viability, with a moderate decrease observed for the 3° RSNO-MSNs (Figure S7). When exposed to 405 nm at any irradiance, significant toxicity to HOECs was observed owing to NO. In contrast, cytocompatibility was noted at 455 nm irradiation for both 400 and 800 mW cm^−2^ (Figure S7). When RSNO-MSNs were incorporated into aBLT, recovery of viability did not occur for any photo-exposure parameters, in contrast to the HPLF cells.

### Planktonic antibacterial activity of photo-initiated NO release

**Figure 3.**
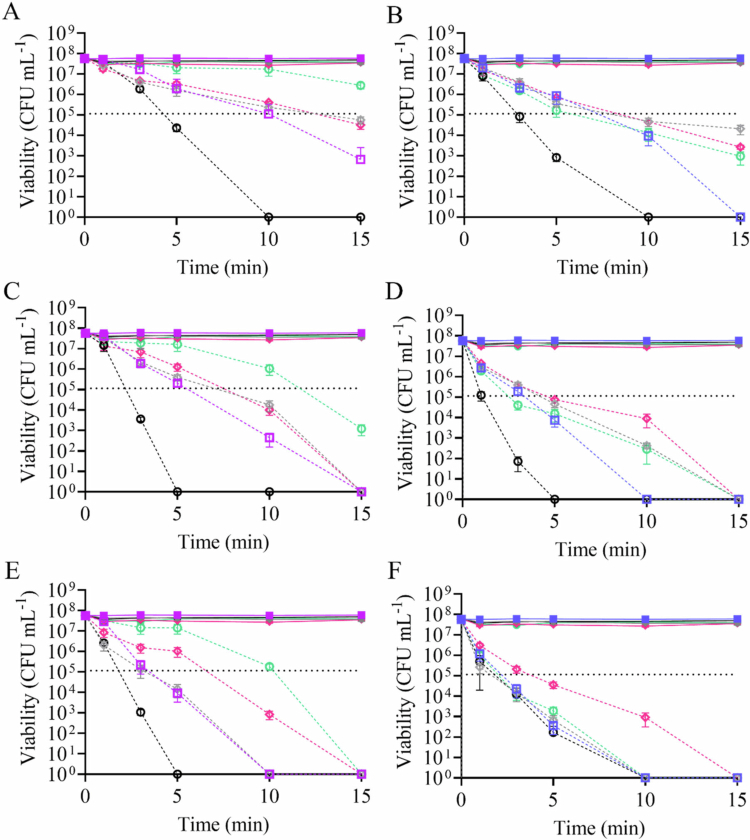
Time-based eradication assay studies of 2 mg mL^−1^ 1° RSNO-MSNs and 3° RSNO-MSNs against *P. gingivalis*. *Porphyromonas gingivalis* was irradiated with (A, C, E) 405 nm or (B, D, F) 455 nm light with irradiances of (A, B) 400 mW cm^−2^, (C, D) 800 mW cm^−2^, or (E, F) 1000 mW cm^−2^. The samples contained no MSNs (solid square), 1° SH-MSNs (grey diamond), 1° RSNO-MSNs (pink diamond), 3° SH-MSNs (black circle), or 3° RSNO-MSNs (green circle). Samples in the dark are solid shapes with solid lines, and samples exposed to LED light are hollow shapes with dashed lines. The horizontal dashed line indicates the 3-log reduction. The error bars represent the standard error of the mean for *n* ≥ 3 biological replicates.

The impacts of aBLT and photo-initiated NO release on *P. gingivalis* were investigated through a time course study wherein aliquots were sampled at intervals between 1 and 15 min. Non-irradiated controls (i.e. 1° SH-MSNs, 1° RSNO-MSNs, 3° SH-MSNs, and 3° RSNO-MSNs) did not exhibit antibacterial activity across all time points (Figure 3). In aBLT (i.e. 405 and 455 nm) alone, 455 nm particles demonstrated greater antibacterial activity than 405 nm (Figure 3). As the irradiance increased, *P. gingivalis* viability decreased for both 405 nm (Figure 3A, 3C, 3E) and 455 nm (Figure 3B, 3D, 3F). The combination of 1 ° SH-MSNs with 405 or 455 nm light did not impact the antibacterial activity. Greater bactericidal action with 3° SH-MSNs was observed for both 405 and 455 nm light (Figure 3). The use of RSNO-MSNs decreased the antibacterial activity of aBLT (when used in combination) against planktonic *P. gingivalis* bacteria. At greater irradiances (i.e. 800 and 1000 mW cm^−2^), a 3-log reduction was achieved for both wavelengths within 15  min, corresponding to NO payloads of approximately 1 µmol mg^−1^ (Figure 3C–3F). As was observed in the cytotoxicity studies above, irradiance impacted pathogen viability. At both wavelengths for all irradiated samples (MSN-containing and non-MSN-containing), increasing irradiance resulted in improved antibacterial action. Given the efficiency of eradication by photo-exposure alone, aBLT and NO combination treatments were not found to be synergistic at 15 min (Table S6).

**Figure 4.**
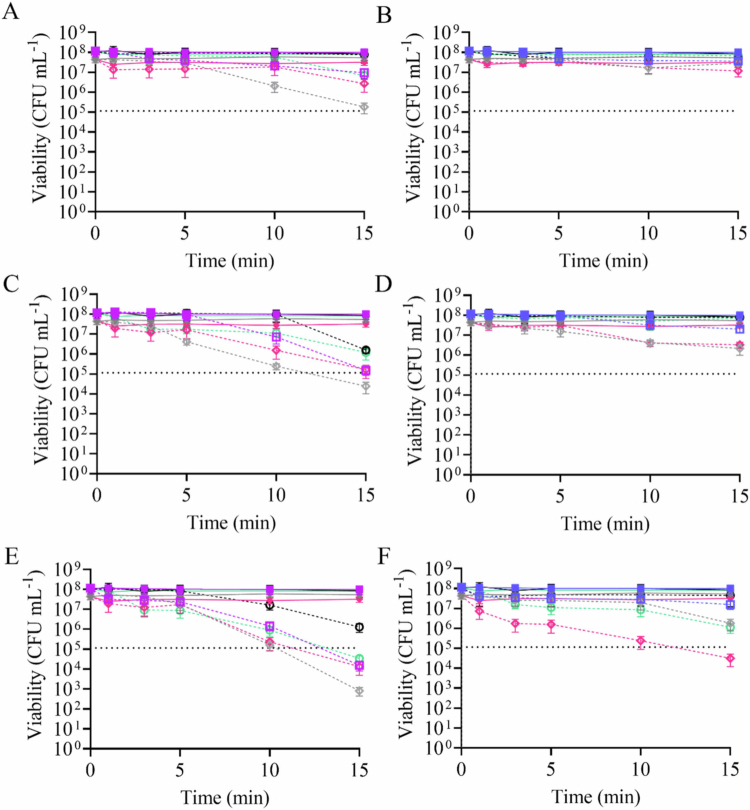
Time-based eradication assay studies of 2 mg mL^−1^ 1° RSNO-MSNs and 3° RSNO-MSNs against *A. actinomycetemcomitans*. *Aggregatibacter actinomycetemcomitans* was irradiated with (A, C, E) 405 nm or (B, D, F) 455 nm light with irradiances of (A, B) 400 mW cm^−2^, (C, D) 800 mW cm^−2^, or (E, F) 1000 mW cm^−2^. The samples contained no MSNs (solid square), 1° SH-MSNs (grey diamond), 1° RSNO-MSNs (pink diamond), 3° SH-MSNs (black circle), or 3° RSNO-MSNs (green circle). The samples in the dark are solid shapes with solid lines, and the samples exposed to LED light are hollow shapes with dashed lines. The horizontal dashed line indicates the 3-log reduction. The error bars represent the standard error of the mean for *n* ≥ 3 biological replicates.

The impact of aBLT with photo-initiated NO release was also investigated against *A. actinomycetemcomitans* using a similar time-course. As seen with *P. gingivalis*, non-irradiated controls (i.e. 1° SH-MSNs, 1° RSNO-MSNs, 3° SH-MSNs, and 3° RSNO-MSNs) did not exhibit antibacterial activity (Figure 4). When exposed to only aBLT, bactericidal action was only observed at the highest irradiance for 405 nm (Figure 4E), with a limited response at 455 nm across all irradiances (Figure 4B, 4D, 4F). With 1° SH-MSNs controls, the antibacterial action of 405 nm across all irradiances was only moderate (Figure 4A, 4C, 4E), with negligible effects at 455 nm (Figure 4B, 4D, 4F). When combined with RSNO-MSNs, a 3-log reduction was observed for 405 nm at 1000 mW cm^−2^ with either RSNO-MSN type (Figure 4E). Only the greatest irradiance (i.e. 1000 mW cm^−2^) at 455 nm was able to achieve a 3-log reduction upon the incorporation of 1° RSNO-MSN, corresponding to approximately 1 µmol mg^−1^. As was observed for *P. gingivalis*, irradiance impacted pathogen viability, with increasing irradiance resulting in improved antibacterial action. Treatments with 405 nm and RSNO-MSNs were largely antagonistic or independent, but treatment with 455 nm and RSNO-MSNs was synergistic in some instances (Table S7). At the greatest irradiance of 1000 mW cm^−2^, treatments with both RSNO-MSN types exhibited synergy, while 1° RSNO-MSNs and 455 nm at 800 mW cm^−2^ also demonstrated synergy.

### Porphyrin extraction and quantification

**Figure 5.**
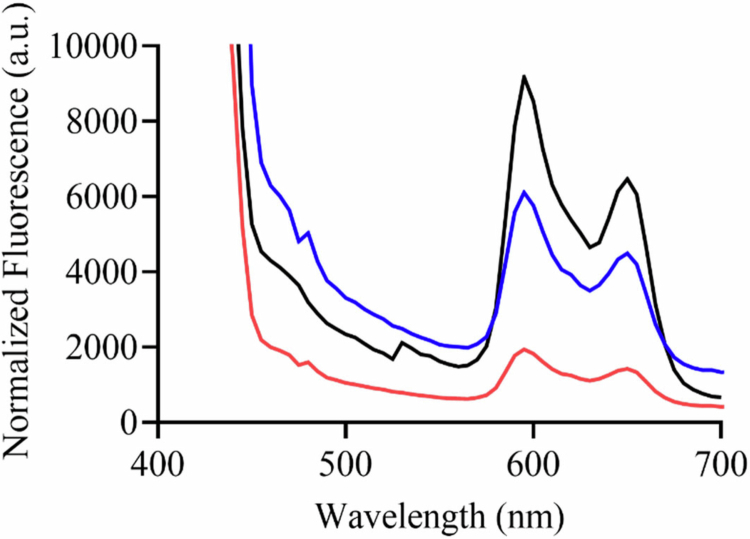
Fluorescence emission of endogenous porphyrin upon 405 nm excitation of *P. gingivalis* (black), *A. actinomycetemcomitans* (blue) and ex vivo biofilm (red).

To better understand the relationship between aBLT and the pathogens of interest, porphyrins were isolated from bacterial cultures of *A. actinomycetemcomitans, P. gingivalis*, and ex vivo subgingival inoculum. The extracted porphyrins were normalized to OD_600_ to elucidate the relative abundances in a given sample. Known as a black pigment-containing pathogen, *P. gingivalis* is known to contain large quantities of available chromophores relative to other bacterial species. Indeed, the levels of extracted porphyrin were greatest in *P. gingivalis*, followed by *A. actinomycetemcomitans*, and the ex vivo plaque biofilm sample (Figure 5), as indicated by increasing fluorescence. The endogenous chromophore content can be correlated with the antibacterial activity of aBLT.

### Anti-biofilm action of photo-initiated NO release

**Figure 6.**
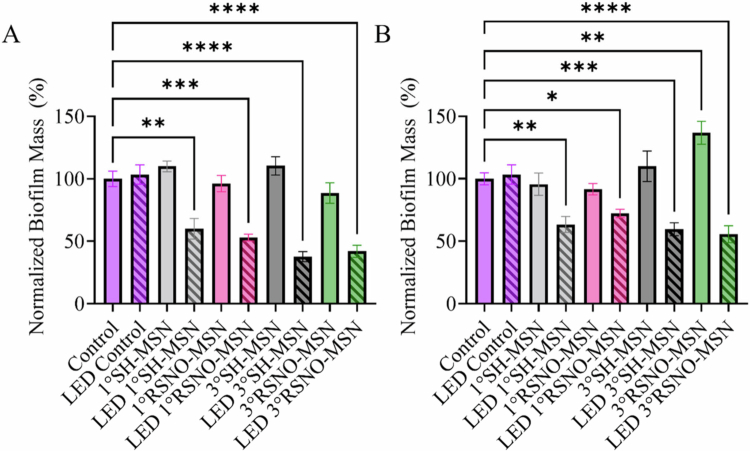
Biofilm dispersal assay of (A) 2 mg mL^−1^ and (B) 8 mg  mL^−1^ MSNs against ex vivo biofilms. The samples were irradiated with 405 nm at 1000 mW cm^−2^ for 15 min, and crystal violet assays were performed 24 h post-treatment. The samples contained no MSNs (purple), 1° SH-MSNs (grey), 1° RSNO-MSNs (pink), 3° SH-MSNs (black), or 3° RSNO-MSNs (green). The samples in the dark are solid bars, and the samples exposed to LED light are striped. The error bars represent the standard error of the mean for *n* ≥ 3 biological replicates.

The antibiofilm activity of aBLT and photo-initiated NO-releasing treatment was assessed at 2 and 8 mg mL^−1^ MSNs to elucidate the impact of the NO concentration on ex vivo biofilm samples. Biofilm dispersion was evaluated 24 h post-treatment with NO-releasing material and 405 nm light at 1000 mW cm^−2^. At 2 mg mL^−1^ MSN, no significant difference in biofilm ability was observed for the SH-MSN or RSNO-MSN controls (Figure 6A). Likewise, photo-exposure alone did not elicit a decrease in biofilm mass. However, a significant decrease in biofilm mass was observed upon the inclusion of all MSN types (i.e. SH-MSN or RSNO-MSN). When MSN concentration was increased to 8 mg mL^−1^, a significant increase in biofilm mass was observed for 3° RSNO-MSNs but did not prove significant for other non-irradiated controls (i.e. 1° SH-MSN, 1° RSNO-MSN, and 3° RSNO-MSN; Figure 6B). When exposed to 405 nm light, MSN-containing samples resulted in significant biofilm dispersion for all MSN types (Figure 6B).

**Figure 7.**
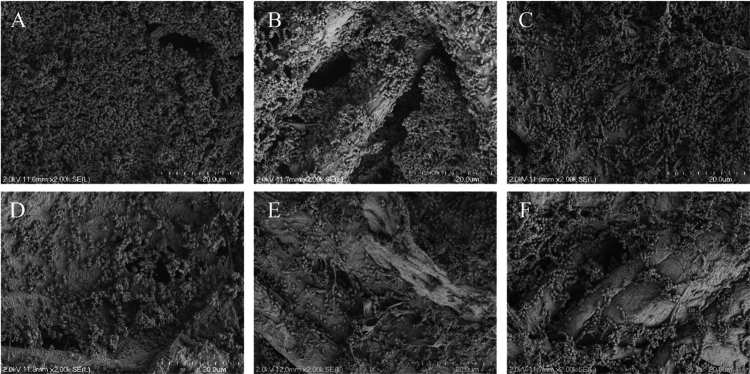
Representative scanning electron micrographs of ex vivo biofilms that were (A) untreated or treated with (B) 1° RSNO-MSNs, (C) 3° RSNO-MSNs, (D) LED, (E) LED 1° RSNO-MSNs and (F) LED 3° RSNO-MSNs. The particle concentration was 8 mg mL^−1^. Scale bars represent 20 μm.

Dispersion of ex vivo biofilms by RSNO-MSNs was visualized using SEM (Figure 7, S8–S10). Non-irradiated controls (i.e. untreated, 1° RSNO-MSN, 3° RSNO-MSNs) were characterized as having dense, multi-layered networks of bacteria, reflecting the structural complexity of the multispecies biofilm. When exposed to 405 nm at 1000 mW cm^−2^ light, a decrease in visualized biofilm was apparent, wherein the EPS matrix was disrupted, and a reduction in bacterial microcolonies was observed compared to non-irradiated controls (Figure 7).

**Figure 8.**
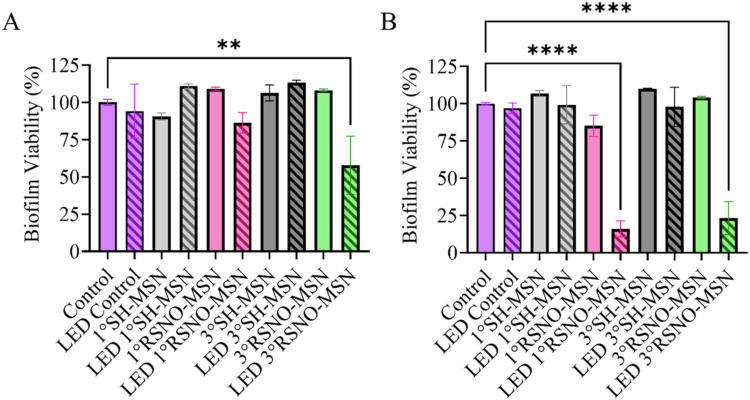
Biofilm viability assay of (A) 2 mg mL^−1^ and (B) 8 mg mL^−1^ MSNs against ex vivo biofilms. The samples were irradiated at 405 nm at 1000 mW cm^−2^ for 15 min, and crystal violet assays were performed 24 h post-treatment. The samples contained no MSNs (purple), 1° SH-MSNs (grey), 1° RSNO-MSNs (pink), 3° SH-MSNs (black), or 3° RSNO-MSNs (green). The samples in the dark are solid bars, and the samples exposed to LED light are striped. The error bars represent the standard error of the mean for *n* ≥ 3 biological replicates.

Biofilm viability was also assessed using resazurin sodium salt over two MSN concentrations. Resazurin reduction was employed as a proxy for cell viability, as the conversion of resazurin to the fluorescent product resorufin is mediated by metabolically active cells. Thus, the assay employed reflects metabolic activity rather than direct enumeration of bacterial colonies. At 2 mg mL^−1^, 1° SH-MSN resulted in a 11% decrease in biofilm metabolic activity, though this decrease was not statistically significant compared to the control (i.e. no treatment), while 3° SH-MSN did not impact biofilm activity at all (Figure 8A). Both types of RSNO-MSNs did not achieve antibacterial activity under native release conditions, but rather slightly increased biofilm metabolic activity by 8–9% (Figure 8A). Photo-irradiation alone (i.e. 405 nm at 1000 mW cm^−2^) did not influence viability, and similarly, photo-irradiation with SH-MSNs did not lead to any changes. The combination of RSNO-MSNs with 405 nm light resulted in a 14 and 42% decrease in biofilm metabolic activity for photo-irradiated 1° RSNO-MSNs and 3° RSNO-MSNs, respectively, with a significant difference observed for the latter, indicating reduced biofilm viability. The particle concentration did not impact the antibacterial activity for photo-irradiated SH-MSNs (8 mg mL^−1^). However, photo-irradiation using 8 mg mL^−1^ RSNO-MSNs resulted in an approximately 80% metabolic decrease, indicating significantly reduced biofilm viability. Photo-initiated NO-releasing treatments were found to be synergistic at both 2 and 8 mg mL^−1^, with greater synergy observed for treatments employing 3° RSNO-MSNs (Table S8).

**Figure 9.**
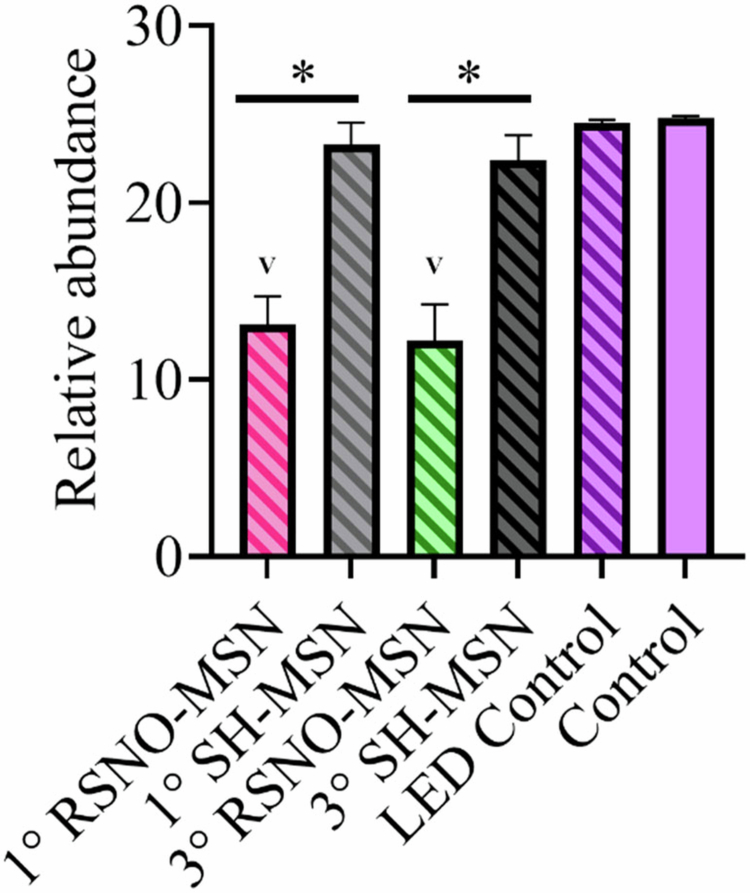
Biofilm bacterial abundance of 8 mg mL^−1^ MSNs against ex vivo biofilms. The samples were irradiated at 405 nm at 1000 mW cm^−2^ for 15 min, and qPCR assays were performed 24 h post-treatment. The samples contained no MSNs (purple), 1° SH-MSNs (grey), 1° RSNO-MSNs (pink), 3° SH-MSNs (black), or 3° RSNO-MSNs (green). The samples in the dark are solid bars, and the samples exposed to LED light are striped. The error bars represent the standard error of the mean for *n* ≥ 3 biological replicates. Significance compared to control and LED control represented by ‘v’.

To further confirm the anti-biofilm action of NO-releasing and photo-irradiation treatments, qPCR was used to assess the remaining bacteria 24 h post-treatment with 8 mg mL^−1^ MSN and 405 nm at 1000 mW cm^−2^. Compared to the non-irradiated control, 1° SH-MSNs and 3° SH-MSNs did not demonstrate significant differences in bacterial abundance (Figure 9). The same was observed against the LED control. When biofilms were treated with the NO-releasing samples (i.e. both 1° RSNO-MSNs and 3° RSNO-MSNs), bacterial abundance significantly decreased compared to both the non-irradiated control and the irradiated control.

## Discussion

The use of aBLT for the eradication of dental pathogens is of great interest, as periodontal disease management requires effective control of complex, multispecies biofilms [24]. While aBLT has potent antibacterial activity, its efficacy is largely dependent on endogenous porphyrin abundance within the bacteria, which varies greatly between species in dental plaque as a result of the heterogeneous nature of the microbial community [49]. Porphyrins readily absorb aBLT, with 405 nm being the most efficacious, as it coincides with the absorption maxima of endogenously produced porphyrin molecules that are more numerous in bacterial cells than in mammalian cells [50,51]. Upon excitation, ROS are generated, leading to oxidative damage to bacterial membranes and intracellular components, thus resulting in antimicrobial activity [52]. The wavelength-specific mechanism allows antibacterial activity to be selectively tuned by modulating photo-exposure parameters (e.g. wavelength, irradiance, duration) to optimize antibacterial activity. To further enhance aBLT therapeutic activity, it may be used in combination with photosensitizers to improve antibacterial activity. However, the use of photosensitizers necessitates repeated treatments to achieve sustained effects [49,53]. Photosensitizers primarily generate ROS, a process that depends on molecular oxygen, which is limited in the anaerobic environment of periodontal biofilms [24]. In contrast, NO has previously demonstrated efficacious antibacterial activity against periodontal pathogens through the production of reactive nitrogen species [54,55]. By harnessing the inherent antibacterial activity of aBLT against porphyrin-containing pathogens with the multi-mechanistic bactericidal nature of NO, a novel therapeutic paradigm may be realized.

Blue light-sensitive NO donors (i.e., RSNOs) were selected for study against periodontal pathogens to assess how the difference in NO-release kinetics and payloads influences antibacterial activity under defined photo-exposure conditions. The facile nature of NO liberation from RSNOs upon photo-irradiation can be further tuned by wavelength, irradiance, and RSNO class (i.e. 1° RSNO-MSNs and 3° RSNO-MSNs), as reported previously [39,56]. By developing a library of NO-release kinetics and NO payloads, a systematic evaluation of photo-initiated release on periodontal pathogens and biofilms becomes achievable. Given that on-demand NO release may influence antimicrobial potency and cellular responses, evaluating cytocompatibility alongside antibacterial activity becomes imperative.

Photo-exposure parameters and NO payloads necessary to treat biofilms may compromise cytocompatibility. Although photo-exposure had the potential to increase the solution temperature by as much as 10 °C, photothermal effects do not result in antibacterial or cytotoxic activity. With aBLT treatment alone, greater irradiances and shorter wavelengths resulted in the greatest cytotoxic effects against both cell lines (i.e., HOECs and HPLFs). Human oral epithelial cells treated with both aBLT and photo-initiated NO release demonstrated greater sensitivity than their HPLF counterparts. In contrast, HPLFs exhibited improved cell viability upon NO photo-delivery compared to LED exposure alone. Differences in the cellular response likely reflect the distinct oxidative and nitrosative stress tolerances between epithelial and fibroblast cell populations. Cells on the outside of the periodontal space (i.e. HOECs) are more sensitive to oxidative stress than fibroblasts (i.e. HPLFs), undergoing cell death in the presence of potent pro-oxidant stimuli [57,58]. Fibroblasts that reside within the periodontal ligament are less sensitive to oxidative stress than HOECs and provide structural support to maintain connective tissue homeostasis [59]. Additionally, improved HPLF cytocompatibility is likely attributed to photon-induced cleavage of the S–N bond within the RSNO moiety, which reduces direct cellular interactions that would otherwise promote ROS-mediated cytotoxicity.

The observed cytotoxicity in HOECs during the treatment window highlights the importance of defining therapeutic windows that balance antibacterial efficacy with host tissue tolerance. Although HOECs did not demonstrate improved cytocompatibility upon photo-induced NO release, monolayer cell cultures do not fully represent the periodontium, where the tissue’s structural complexity, cellular interactions, and diffusion dynamics may attenuate the response to photo-induced NO release and aBLT. Moreover, the oral epithelium exhibits rapid surface turnover (i.e. cell renewal within hours), allowing for damaged cells to be quickly replaced to prevent the accumulation of photo- or NO-induced cellular damage within the tissue [60]. Scattering and photon absorption by crevicular fluid in the periodontal pocket are expected to additionally reduce the effective irradiance reaching target tissues, which has the potential to mitigate cytotoxic effects and expand the effective treatment window relative to uniform in vitro exposure. Further, in vitro conditions represent a simplified exposure scenario that does not capture the effects of localized irradiation or potential approaches to mitigate cytotoxicity in vivo. In the context of photo-based therapeutics for the periodontium, clinical translation may be facilitated by fibre optics or probe-based light delivery, allowing for enhanced, localized treatment of pathogenic biofilms while minimizing exposure to surrounding tissues [61]. Strategies to enhance tissue tolerance include reducing fluence, introducing pulsed light (i.e. duty cycle) to mitigate toxic byproducts like ROS, and treating periodically rather than single-time treatments.

While mammalian cells can be susceptible to high ROS levels, bacteria are inherently less equipped to mitigate oxidative and nitrosative stress, rendering them more sensitive to photo-initiated NO release and aBLT-mediated damage [22,62]. Two key periodontal disease pathogens, *P. gingivalis* and *A. actinomycetemcomitans,* have proven susceptible to aBLT and NO treatments independently, but the combined effect of these modalities has yet to be investigated [63–65]. In the planktonic state, aBLT was efficacious against both *P. gingivalis* and *A. actinomycetemcomitans,* with greater susceptibility observed for *P. gingivalis.* As an obligate anaerobe, *P. gingivalis* will have a less robust antioxidant defence system than the facultative anaerobe *A. actinomycetemcomitans,* which is apparent upon exposure to aBLT [66]. Interestingly, 3° SH-MSNs exposed to aBLT presented the most rapid decrease in antibacterial activity against *P. gingivalis.* Owing to the nature of the 3° SH moiety, it is expected that cellular interactions with the 3° SH-MSNs may increase relative to the 1° SH-MSNs due to changes in charge and hydrophilic characteristics. The hydrophilic nature of the 3° SH-MSNs results in a stronger association with the bacterial membrane of *P. gingivalis* [37]. Potent antibacterial activity was observed because of the light scattering afforded by the MSNs.

In photo-initiated NO-release studies, a 3-log reduction was achieved in nearly all NO-releasing samples across both wavelengths and three irradiance profiles against *P. gingivalis.* Incorporating RSNO-MSNs slowed the antibacterial activity against *P. gingivalis*. This effect was likely the result of photon absorption across multiple photo-active species (e.g. RSNO and chromophores) as opposed to endogenous porphyrinsalone. As such, ROS production is likely decreased, corresponding to greater pathogen viability. Furthermore, the rate of bactericidal action against *P. gingivalis* was impacted by the RSNO-MSN type when exposed to 405 nm light, corresponding to changes in NO-release kinetics. The large NO flux from 1 ° RSNO-MSNs afforded by 405 nm exposure resulted in more rapid antibacterial activity than 3° RSNO-MSNs. In contrast, when exposed to 455 nm light, sustained NO release by 3° RSNO-MSNs resulted in faster antibacterial activity, where NO payloads (~1 µmol mg^−1^) governed antibacterial action as opposed to NO-release kinetics. In contrast, for *A. actinomycetemcomitans* studies, inclusion of NO photo-release did not hinder 405 nm activity and was necessary for 455 nm to achieve antibacterial activity. At both wavelengths, 1° RSNO-MSNs demonstrated similar or improved efficacy over 3° RSNO-MSNs. It is imperative to note that while comparable antibacterial activity was achieved for aBLT and photo-initiated NO release across planktonic pathogens, biofilm dispersal and eradication is far more challenging.

Biofilm dispersal is critical for effective therapeutic treatment of periodontal disease. Patient-derived biofilms offer microbial diversity and structural organization typical of subgingival biofilms, providing a model that more closely reflects the periodontium. While the 24 h growth period used in the above studies may not fully capture the complexity and resilience of mature biofilms, this system nonetheless provides a valuable platform for evaluating photo-based therapies and informs future studies aimed at optimizing efficacy. Low NO levels delivered by non-irradiated RSNO-MSNs resulted in a slight decrease in biofilm mass, albeit insignificantly, which is consistent with NO-mediated biofilm dispersal signalling initiated by cyclic-di-GMP modulation [67]. In contrast, 3° RSNO-MSNs at 8 mg mL^−1^ led to a significant increase in biofilm, which may be attributed to a variety of factors, including increased nitrogen availability derived from 3° RSNO-MSN functionalization, facile reformation of the RSNO moiety due to the high MSN concentration, or increased EPS production as a protective biofilm response. Notably, NO has been reported to exhibit concentration-dependent effects on both dispersing and promoting biofilm formation [68]. Although these findings warrant further mechanistic investigation, they underscore the importance of controlled NO delivery in achieving the desired antibiofilm effect. Accordingly, the proposed treatment strategy leverages aBLT and on-demand NO release to modulate dosing and enhance treatment efficacy.

As previously observed, aBLT is effective against planktonic periodontal pathogens, but has been reported to have limited utility against periodontal biofilms without an adjuvant agent [69,70]. By combining aBLT with on-demand NO release at two RSNO-MSN concentrations (i.e. 2 and 8 mg mL^−1^), biofilm penetration and dispersal may be possible. When ex vivo periodontal biofilms were exposed to 405 nm at 1000 mW cm^−2^ alone, dispersion was not observed. However, the addition of SH-MSNs and RSNO-MSNs to aBLT resulted in significant biofilm dispersal. The incorporation of MSNs scattered incident light evenly across the biofilm, thereby increasing the photon residence time and improving dispersal. When visualized with SEM, structural observations confirm that photo-activated RSNO-MSNs can effectively decrease adhered biofilm mass, highlighting the potential of on-demand NO release as a complementary antibacterial therapy to aBLT.

While periodontal biofilm dispersal is important to facilitate effective periodontal disease treatment, dispersal without pathogen eradication can worsen infection, wherein residual microcolonies can regrow once therapeutic treatment is halted [71]. Although photo-exposure by 405 nm light at 1000 mW cm^−2^ proved bactericidal against planktonic pathogens, no significant antibiofilm activity was observed against ex vivo biofilms. The addition of photo-initiated NO release was crucial for effective antibiofilm activity. When treated with 2 mg mL^−1^, antibiofilm activity was governed by NO-release kinetics where the rapid NO flux afforded by 1° RSNO-MSNs did not result in significant decreases in biofilm viability, as determined by metabolic activity through a resazurin-based assay. However, the sustained NO release of photo-irradiated 3° RSNO-MSNs allowed for efficient biofilm penetration and subsequent antibiofilm activity compared to the untreated control. Although the NO payloads were comparable at ~1 µmol mg^−1^ for both RSNO-MSN types, NO-release kinetics greatly varied. This finding underscores the importance of NO-release kinetics in addition to the NO payload in determining antibacterial efficacy. When the RSNO-MSN concentration was increased to 8 mg mL^−1^, ex vivo biofilm metabolic activity decreased significantly upon photo-initiated NO-release from both 1° RSNO-MSNs and 3° RSNO-MSNs, indicating reduced biofilm viability. The equivalent antibacterial effects suggest that a sufficiently large NO flux from 1° RSNO-MSNs can overcome the advantages of sustained NO release attributed to the 3° RSNO-MSNs. These results are further corroborated by the bacterial abundance, wherein 1° RSNO-MSNs and 3° RSNO-MSNs at 8 mg mL^−1^ resulted in significant bacterial reduction when irradiated with 405 nm at 1000 mW cm^−2^. While the analysis in this work was limited to total bacterial abundance without species-level resolution, the model provides a valuable platform for evaluating the efficacy of photo-based therapeutics under physiologically relevant conditions. To this end, NO is essential to overcome the protective mechanisms of dental biofilms.

## Conclusion

The combination of aBLT and photo-initiated NO release represents a highly effective strategy for combating periodontal pathogens, particularly within resilient biofilm communities. While aBLT proved effective against planktonic bacteria, biofilm disruption and eradication did not occur, highlighting the critical role of on-demand NO delivery as an adjuvant therapeutic. Nitric oxide payloads and release kinetics are key determinants of antibiofilm efficacy, wherein 2 mg mL^−1^ of RSNO-MSNs necessitated sustained release to achieve significant effects, whereas higher NO payloads afforded by 8 mg mL^−1^ overcame kinetic limitations, offering flexibility in therapeutic design. Importantly, the combination treatment of aBLT and photo-initiated NO release significantly improved the viability of HPLFs, suggesting that antimicrobial efficacy can be enhanced without compromising host cell health. These findings underscore the need for careful balancing of NO delivery parameters to maximize therapeutic outcomes while minimizing cytotoxicity. Collectively, this work provides a mechanistic and practical framework for the rational design of next-generation light-activated NO therapies, offering a promising approach for the treatment of periodontal disease.

## Supplementary Material

Supplementary Material Supporting_Info_V5_Submission 2.pdf

## Associated content

Supporting Data. Materials and methods for MSN and SH-MSN synthesis, MBC assay, and preliminary cytotoxicity assay; CHNS elemental analysis of MSNs and SH-MSNs; total NO loading for RSNO-MSNs; photolytic NO maximum; quantum yield of photo-initiated NO release; MBC results against *A. actinomycetemcomitans* and *P. gingivalis*; evaluation of synergy for treatments against *A. actinomycetemcomitans, P. gingivalis,* and ex vivo biofilms; SEM images of MSN and SH-MSNs; cytotoxicity curves of HOECs and HPLFs; temperature change upon photo-exposure; photothermal bacterial and cell viability; photo-toxicity of HOECs; SEM of treated ex vivo biofilm; representative CFU plates.
