# Supplementary material for: Treatment of periodontal biofilms via nitric oxide-augmented phototherapy
Source: J Oral Microbiol. 2026 Apr 28;18(1):2658899. doi: 10.1080/20002297.2026.2658899 (PMC13126946; doi:10.1080/20002297.2026.2658899)
Supplement: Supplementary Material — Supporting_Info_V5_Submission 2.pdf [file ZJOM_A_2658899_SM3741.pdf]

Treatment of Periodontal Biofilms Via Nitric Oxide-Augmented Phototherapy

Courtney R. Johnson[1], *Tsian D. Ramrattan*[1], *Casimir J. Schaefer*[1], *Rajnish Kumar*[1], *Simon J. Reinhard*[2], *Shannon M. Wallet*[2], *and Mark H. Schoenfisch*[1,3]

[1]Department of Chemistry, University of North Carolina at Chapel Hill, Chapel Hill, NC 27599

[2]Department of Oral Biology, College of Dentistry, University of Florida, Gainesville, Florida 32610, United States.

[3]Eshelman School of Pharmacy, University of North Carolina at Chapel Hill, Chapel Hill, NC 27599

* Corresponding Author: schoenfisch@unc.edu

Table of Contents:

1. MATERIALS AND METHODS	S3-S4
2. **Table S1.** CHNS	S5
3. **Table S2.** Total NO Loading on RSNO-MSNs	S5
4. **Table S3.** Instantaneous NO Maximum Upon Photolysis	S6
5. **Table S4.** Initial Quantum Yield for NO Release Upon Photolysis	S7
6. **Table S5.** MBC for *A. actinomycetemcomitans* and *P. gingivalis*	S7
7. **Table S6.** Evaluation of Synergistic Treatment Interactions Against *P. gingivalis*	S8
8. **Table S7.** Evaluation of Synergistic Treatment Interactions Against *A. actinomycetemcomitans*	S8
9. **Table S8.** Evaluation of Synergistic Treatment Interactions Against Ex Vivo Periodontal Biofilms	S9
10. **Figure S1.** SEM of MSNs	S10
11. **Figure S2.** LED Experimental Scheme	S10

12. **Figure S3.** Cell Viability Curves for HOECs and HPLFs	S11
13. **Figure S4.** Temperature Change Upon Photo-Exposure	S11
14. **Figure S5.** Impact of Photothermal Heating on Planktonic Pathogens	S12
15. **Figure S6.** Impact of Photothermal Heating on HOECs	S12
16. **Figure S7.** Photo-Toxicity of HOECs	S13
17. **Figure S8.** SEM of Treated Ex Vivo Biofilm (2 mg mL^{-1} MSNs)	S14
18. **Figure S9.** SEM of Treated Ex Vivo Biofilm (2 & 8 mg mL^{-1} MSNs) – Low magnification	S15
19. **Figure S10.** SEM of Treated Ex Vivo Biofilm (2 & 8 mg mL^{-1} MSNs) – High magnification	S16
20. **Figure S8.** Representative CFU Plates	S17

MATERIALS AND METHODS

Materials.

Particle Characterization.

Particle morphology and size were determined using scanning electron microscopy (SEM). Particles were coated in a 10.0 nm layer of Au/Pd prior to imaging on a Hitachi S-4700 cold cathode field emission scanning electron microscope (Pleasanton, CA). Thiol quantification for MSNs and SH-MSNs was completed using carbon, hydrogen, nitrogen, sulfur (CHNS) elemental analysis where a PerkinElmer Elemental Analyzer Series 2400 instrument was employed (Waltham, MA).

Minimum Bactericidal Concentration Assay.

Bacteria grown to the log phase, as mentioned previously, were diluted 5 x 10^5 CFU mL^{-1} in 10% BHI or WCB and 100 μL added per well in a 96-well plate for minimum bactericidal concentration (MBC) experiments. Bacteria were challenged against 1° SH-MSNs, 3° SH-MSNs, 1° RSNO-MSNs, and 3° RSNO-MSNs ranging from 0.03 to 16 mg mL^{-1} in a 2-fold dilution series for 24 h at 37°C in anaerobic conditions. After 24 h, an aliquot was taken from each well (10 μL), plated on BHI or supplemented WCA, and grown for 48-72 h. The MBC was determined by recording the dilution at which no bacterial growth was observed.

In Vitro Cytotoxicity.

Upon achieving 80% confluency, cells (i.e., HOEC or HPLF) were seeded in polystyrene 96-well plates at 5 x 10^5 cells mL^{-1} and allowed 24 h of incubation to adhere. After 24 h, media was aspirated from the wells and replaced with fresh media and 1° SH-MSNs, 3° SH-MSNs, 1°

RSNO-MSNs, or 3° RSNO-MSNs, in a 2-fold serial dilution from 0.03-16 mg mL^{-1} for 24 h treatment. Cells were then treated with resazurin sodium salt solution (20 µL of 0.1 mg mL^{-1} per well), incubated 4 h, and fluorescence measured at 544 nm excitation and 590 nm emission wavelengths using a Molecular Devices SpectraMax M2 spectrophotometer (San Jose, CA). Cell viability was calculated by Equation 2. Fresh media was used as the blank and non-treated cells served as controls.

Table S1. CHNS Elemental Analysis of MSNs and SH-MSNs[a]

Nanoparticle	%C	%H	%N	%S
MSN	1.66 ± 0.40	1.44 ± 0.13	0.01 ± 0.01	0.20 ± 0.40
1° SH-MSN	12.22 ± 1.53	2.40 ± 0.32	0.20 ± 0.04	6.68± 0.37
3° SH-MSN	15.32 ± 0.61	2.96 ± 0.20	3.33 ± 0.22	4.30 ± 1.63

[a]Error represents the standard deviation for n ≥ 3 syntheses

Table S2. Total NO Loading on RSNO-MSNs[a]

Nanoparticle	NO_{total} (μmol mg^{-1}) [b]
1° RSNO-MSN	1.24 ± 0.05
3° RSNO-MSN	0.98 ± 0.02

[a]Error represents the standard deviation for n ≥ 3 analyses.
[b]Total NO released upon addition of $CuBr_2$ (2.5 mM)

Table S3. Instantaneous NO Maximum Upon Photolysis[a]

**Wavelength (nm)**	**Irradiance ($mW\ cm^{-2}$)**	**NO_{max} ($pmol\ mg^{-1}$)[b]**
	Primary RSNO	
Native	No Light	146 ± 69
405	400	6651 ± 1147
	800	8759 ± 912
	1000	7933 ± 76
455	400	2135 ± 280
	800	2751 ± 615
	1000	3490 ± 137
	Tertiary RSNO	
Native	No Light	29 ± 16
405	400	2485 ± 312
	800	4033 ± 729
	1000	4280 ± 629
455	400	409 ± 27
	800	720 ± 14
	1000	880 ± 153

[a]Error represents the standard deviation for n ≥ 3 analyses
[b]Total NO released upon photo-irradiation.

Table S4. Initial Quantum Yield for NO Release Upon Photolysis

Nanoparticle	Wavelength (nm)	Irradiance (mW cm^{-2})	Quantum Yield (%)
1° RSNO-MSN	405	400	0.348 ± 0.051
		800	0.220 ± 0.037
		1000	0.132 ± 0.028
	455	400	0.091 ± 0.011
		800	0.058 ± 0.017
		1000	0.056 ± 0.006
3° RSNO-MSN	405	400	0.093 ± 0.024
		800	0.060 ± 0.009
		1000	0.056 ± 0.008
	455	400	0.018 ± 0.003
		800	0.017 ± 0.003
		1000	0.016 ± 0.002

Table S5. Minimum Bactericidal Concentration (MBC) and Bactericidal NO Dose of MSNs Against *A. actinomycetemcomitans* and *P. gingivalis* Following 24 h Exposure[a]

	Aggregatibacter actinomycetemcomitans		***Porphyromonas gingivalis***	
**Treatment**	**MBC (mg mL^{-1})**	**MBC_{NO} (µg mL^{-1})**	**MBC (mg mL^{-1})**	**MBC_{NO} (µg mL^{-1})**
1° SH-MSN	>16	-	>16	-
1° RSNO-MSN	4	49	2	25
3° SH-MSN	>16	-	>16	-
3° RSNO-MSN	>16	>69	8	35

[a]MBC 24 h determined from n ≥ 3 biological replicates. [b]NO dose derived from the MBC 24 h and the total NO released over the 24 h exposure time in PBS (10 mM, pH 7.4, 37 °C)

Table S6. Evaluation of Synergistic Interactions Against *P. gingivalis*

Nanoparticle	Wavelength (nm)	Irradiance (mW cm^{-2})	Bliss Independence ($\Delta Y_{LED/NO}$)[a]
1° RSNO-MSN	405	400	0.00
		800	0.00
		1000	0.00
	455	400	0.00
		800	0.00
		1000	0.00
3° RSNO-MSN	405	400	-0.05
		800	0.00
		1000	0.00
	455	400	0.00
		800	0.00
		1000	0.00

[a]Synergy is $\Delta Y_{LED/NO} > 0$, antagonistic is $\Delta Y_{LED/NO} < 0$, and independent is $\Delta Y_{LED/NO} = 0$.

Table S7. Evaluation of Synergistic Interactions Against *A. actinomycetemcomitans*

Nanoparticle	Wavelength (nm)	Irradiance (mW cm^{-2})	Bliss Independence ($\Delta Y_{LED/NO}$)[a]
1° RSNO-MSN	405	400	-0.02
		800	-0.06
		1000	-0.08
	455	400	-0.04
		800	0.05
		1000	0.10
3° RSNO-MSN	405	400	0.00
		800	-0.01
		1000	0.00
	455	400	-0.41
		800	-0.52
		1000	0.09

[a]Synergy is $\Delta Y_{LED/NO} > 0$, antagonistic is $\Delta Y_{LED/NO} < 0$, and independent is $\Delta Y_{LED/NO} = 0$.

Table S8. Evaluation of Synergistic Interactions Against Ex Vivo Periodontal Biofilms

Wavelength (nm)	Irradiance (mW cm^{-2})	Nanoparticle	Bliss Independence ($\Delta Y_{LED/NO}$)[a]
405	1000	1° RSNO-MSN (2 mg mL^{-1})	0.16
		1° RSNO-MSN (8 mg mL^{-1})	0.25
		3° RSNO-MSN (2 mg mL^{-1})	0.86
		3° RSNO-MSN (8 mg mL^{-1})	0.78

[a]Synergy is $\Delta Y_{LED/NO} > 0$, antagonistic is $\Delta Y_{LED/NO} < 0$, and independent is $\Delta Y_{LED/NO} = 0$.

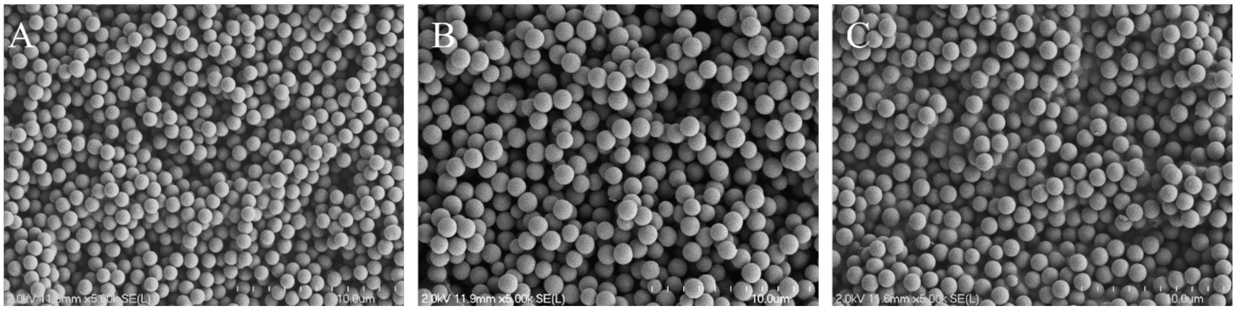

Figure S1. Representative SEM images of A) MSNs and B) 1° SH-MSNs, C) 3° SH-MSNs. Scale bars represent 5 μm. Particles maintain spherical morphology following surface grafting.

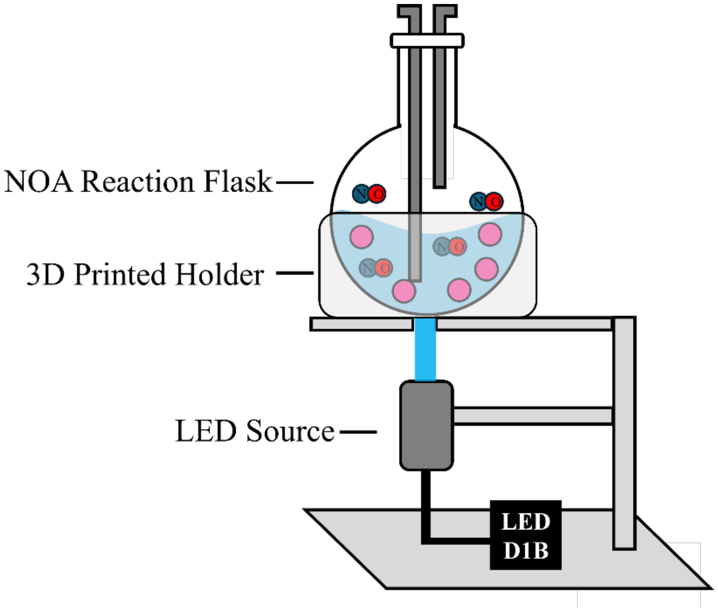

Figure S2. LED experimental scheme. LED light is positioned directly below stage onto which the NOA reaction flask or quartz crucible rests. The NOA reaction flask is held in place with a custom 3D printed holder. A 12.5 mm diameter hole is present in the stage for light exposure.

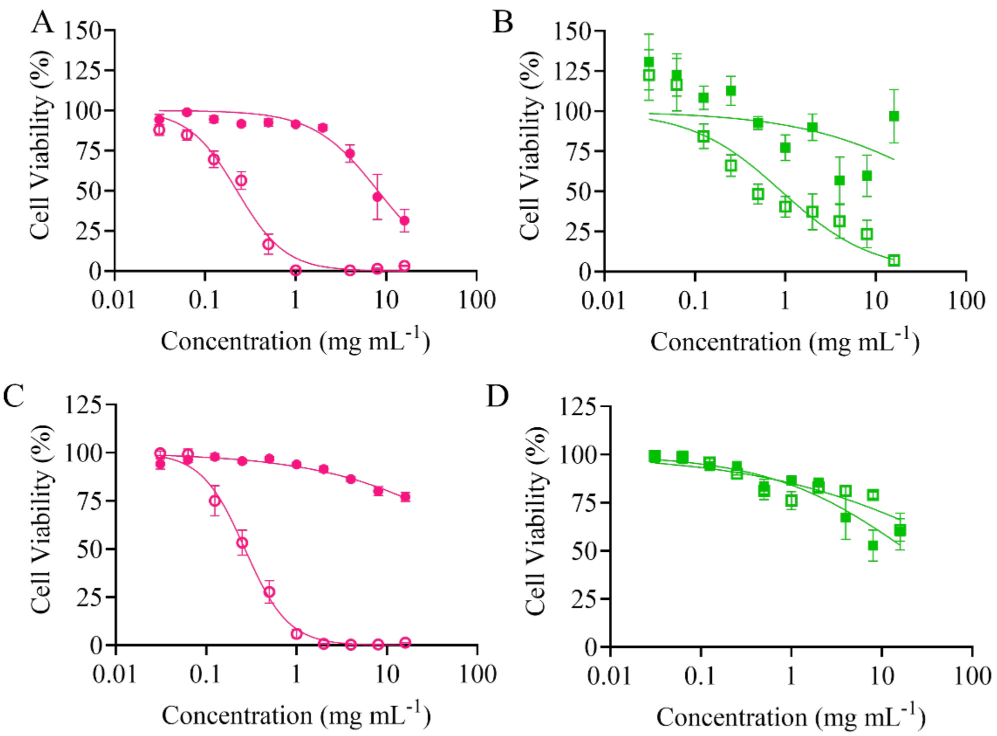

Figure S3. Cytotoxicity curves of A, B) HOEC and C, D) HPLF cells against A, C) 1°RSNO-MSNs or B, D) 3° RSNO-MSNs. Error bars represent standard error of the mean for n ≥ 3 biological replicates.

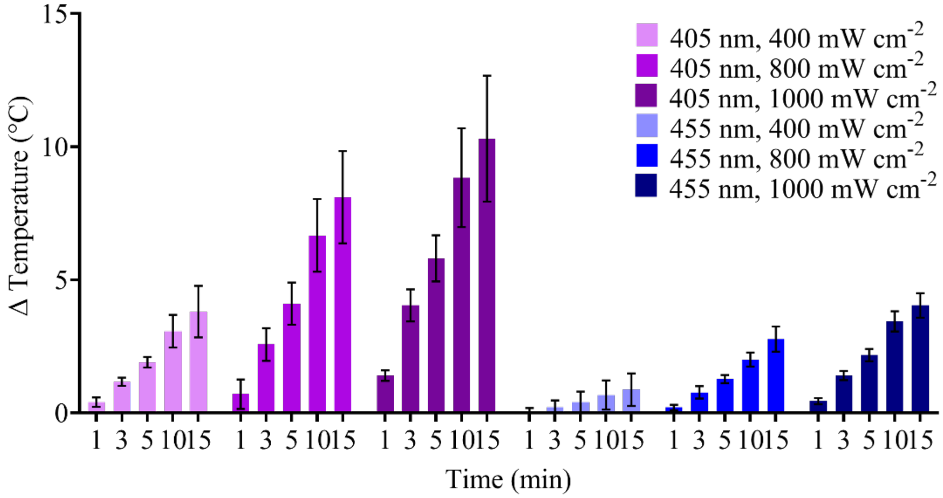

Figure S4. Change in temperature of 1 mL PBS upon aBLT exposure. Error is represented by standard deviation for n ≥ 3 replicates.

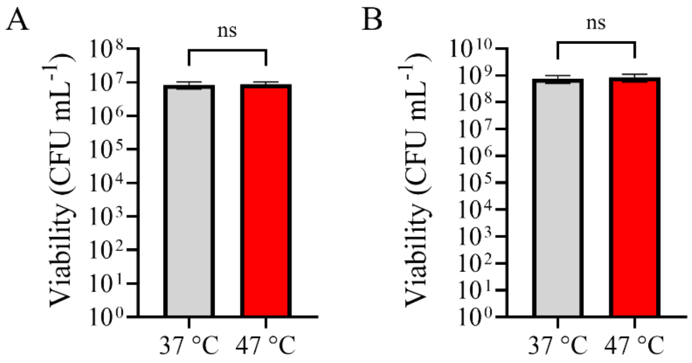

Figure S5. Antibacterial activity of A) *P. gingivalis* and B) *A. actinomycetemcomitans* following exposure to photothermal-induced solution temperatures. Error bars represent standard error of the mean for n ≥ 3 biological replicates.

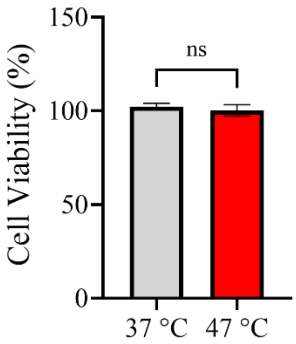

Figure S6. Cell viability of HOEC following exposure to photothermal-induced solution temperatures. Error bars represent standard error of the mean for n ≥ 3 biological replicates.

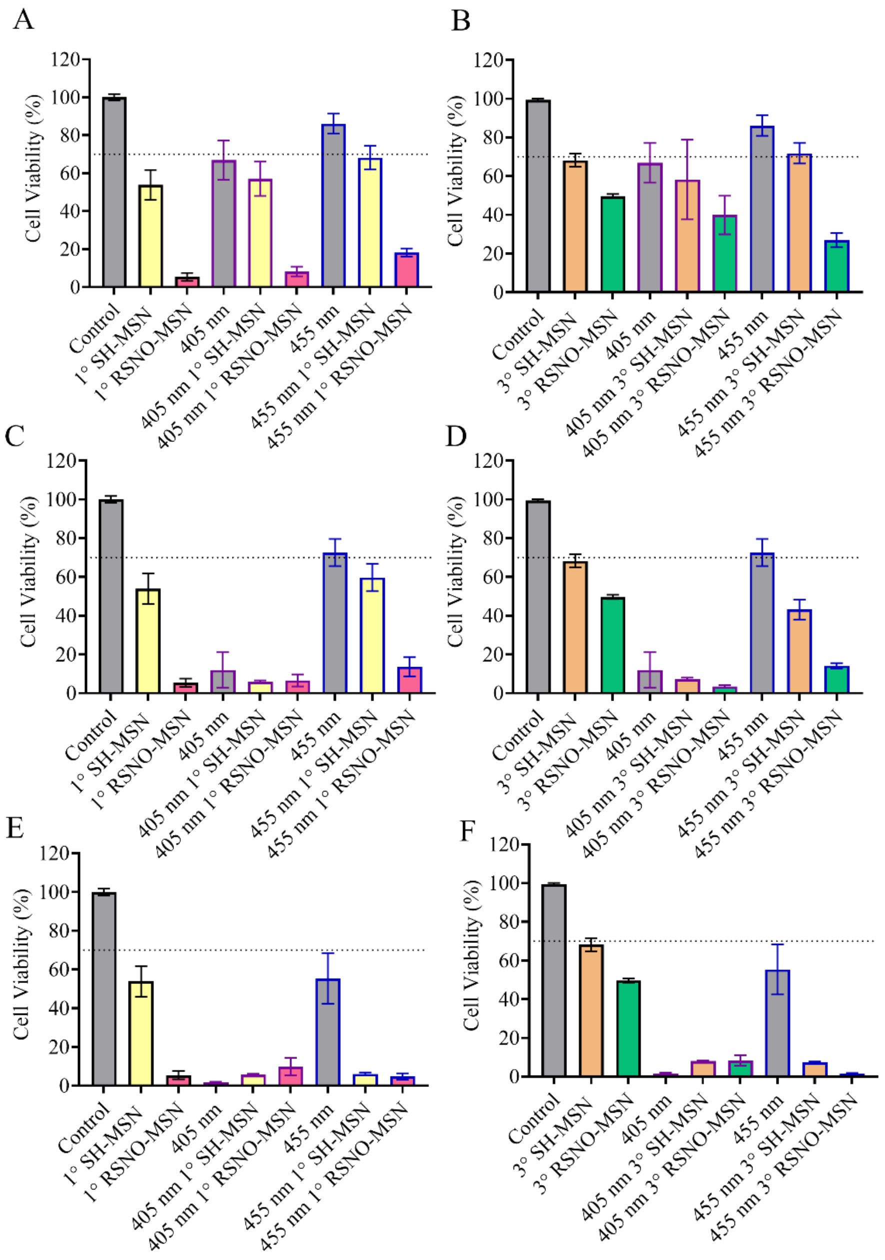

Figure S7. Photo-toxicity studies of 2 mg mL^{-1} 1° RSNO-MSNs and 3° RSNO-MSNs 24 h post treatment against HOEC. Cells were irradiated with A, C, E) 405 nm or B, D, F) 455 nm light with irradiances of A, B) 400 mW cm^{-2}, C, D) 800 mW cm^{-2}, or E, F) 1000 mW cm^{-2}. Samples contained no MSNs (gray), 1° SH-MSNs (yellow), 1° RSNO-MSNs (pink), 3° SH-MSNs (orange), or 3° RSNO-MSNs (green). The dashed line indicates 70% viability (i.e., cytocompatible). Error bars represent standard error of the mean for $n \geq 3$ biological replicates.

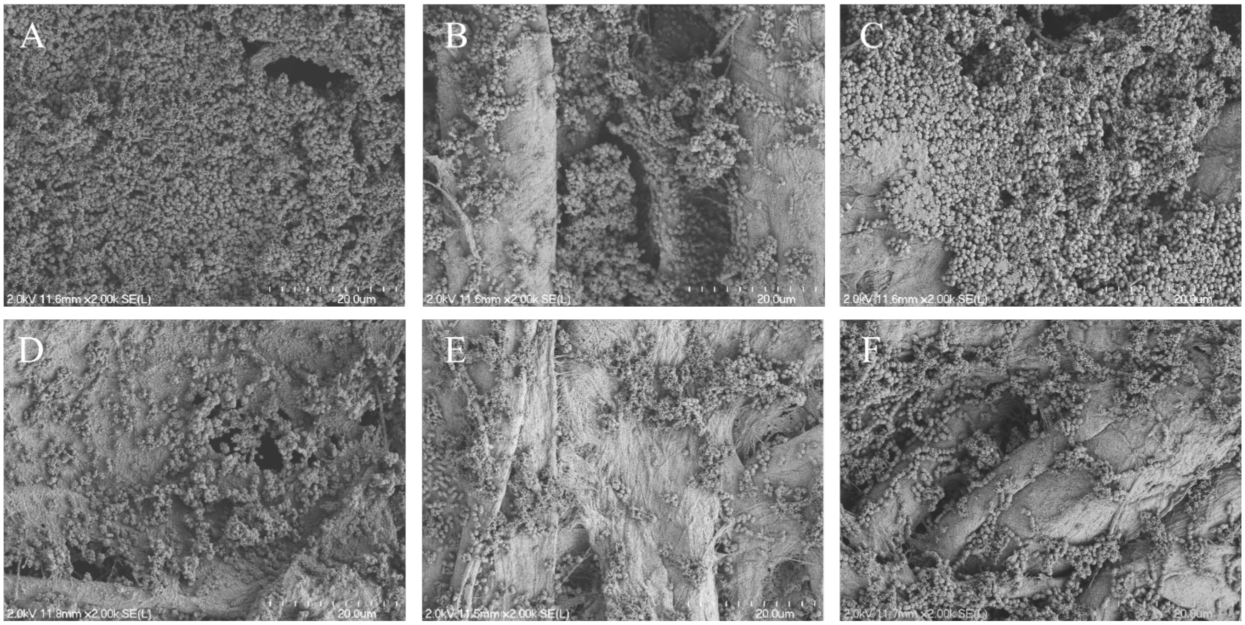

Figure S8. Representative scanning electron micrographs of ex vivo biofilms that were A) untreated or treated with B) 1° RSNO-MSNs, C) 3° RSNO-MSNs, D) LED, E) LED 1° RSNO-MSNs, and F) 3° RSNO-MSNs. Particle concentration is 2 mg mL^{-1}. Scale bars represent 20 μm.

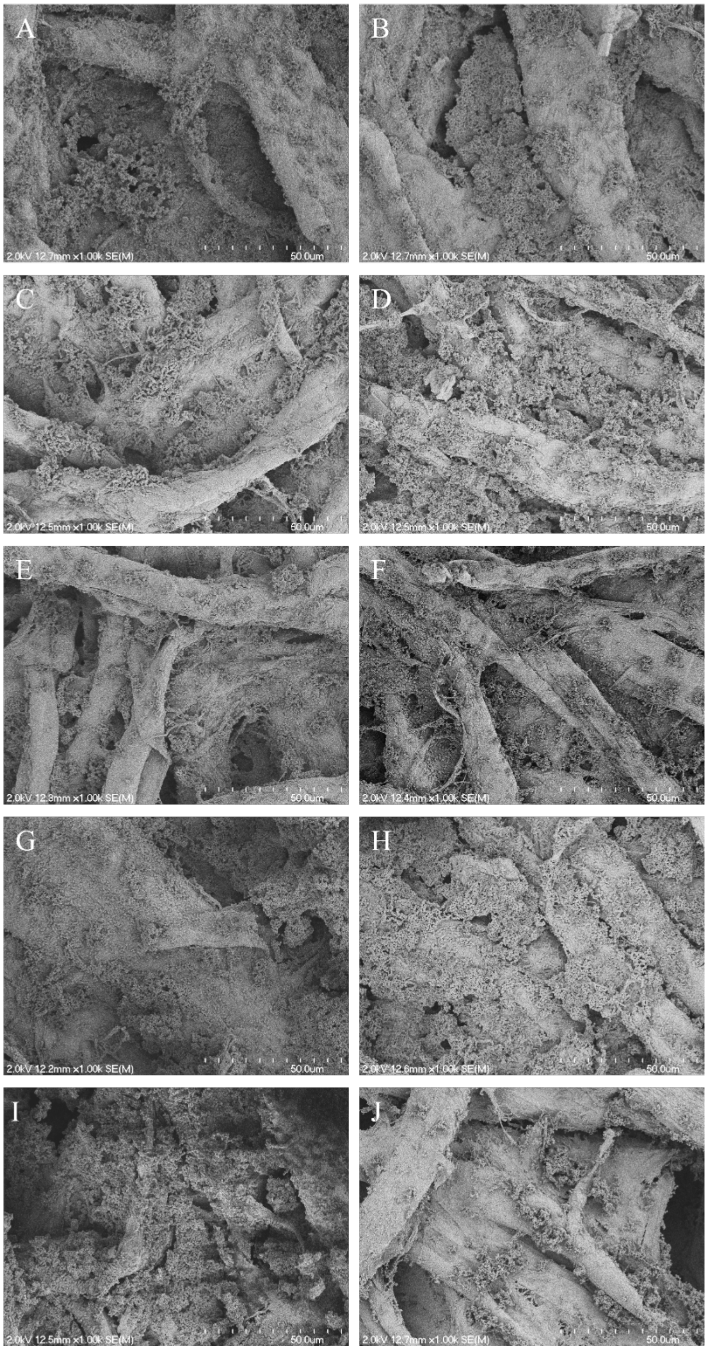

Figure S9. Representative scanning electron micrographs of ex vivo biofilms that were A) untreated or treated with B) LED, C) 1° RSNO-MSNs (2 mg mL^{-1}), D) LED 1° RSNO-MSNs (2 mg mL^{-1}), E) 1° RSNO-MSNs (8 mg mL^{-1}), F) LED 1° RSNO-MSNs (8 mg mL^{-1}), G) 3° RSNO-MSNs (2 mg mL^{-1}), H) LED 3° RSNO-MSNs (2 mg mL^{-1}), I) 3° RSNO-MSNs (8 mg mL^{-1}), J) LED 3° RSNO-MSNs (8 mg mL^{-1}). Scale bars represent 50 μm.

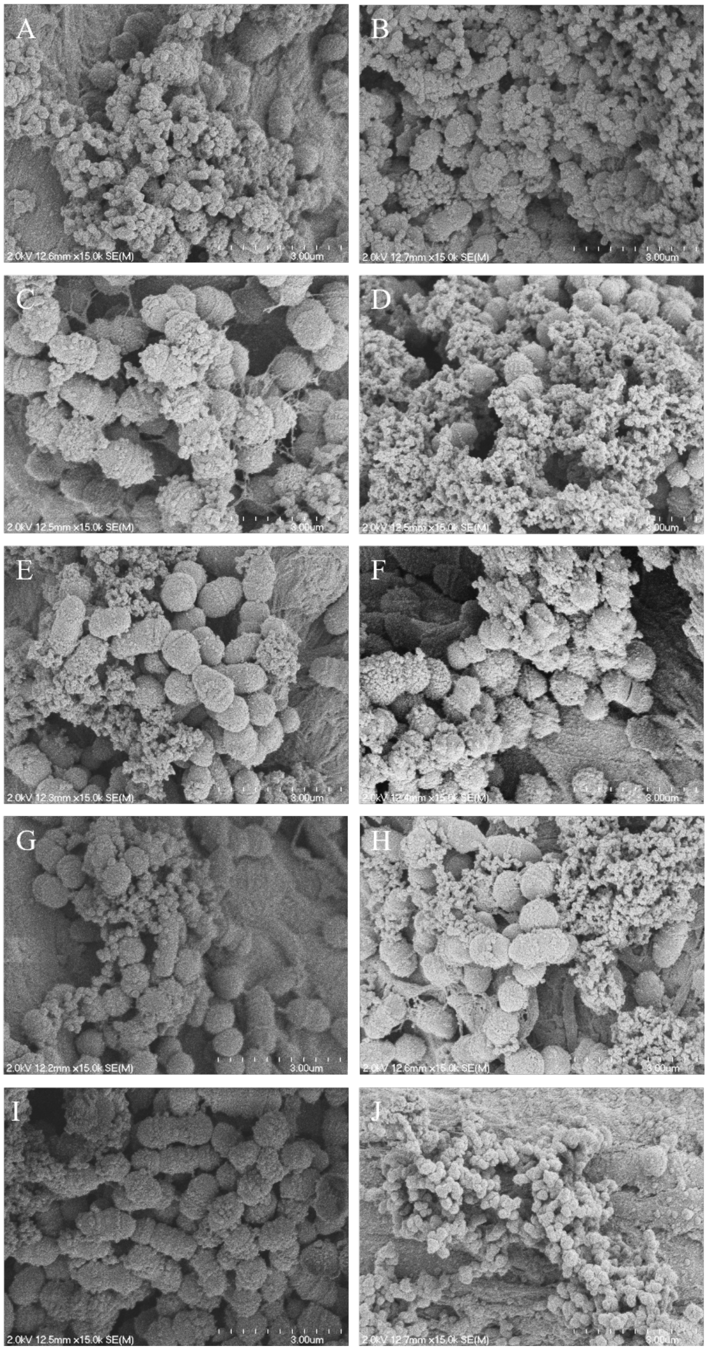

Figure S10. Representative scanning electron micrographs of ex vivo biofilms that were A) untreated or treated with B) LED, C) 1° RSNO-MSNs (2 mg mL^{-1}), D) LED 1° RSNO-MSNs (2 mg mL^{-1}), E) 1° RSNO-MSNs (8 mg mL^{-1}), F) LED 1° RSNO-MSNs (8 mg mL^{-1}), G) 3° RSNO-MSNs (2 mg mL^{-1}), H) LED 3° RSNO-MSNs (2 mg mL^{-1}), I) 3° RSNO-MSNs (8 mg mL^{-1}), J) LED 3° RSNO-MSNs (8 mg mL^{-1}). Scale bars represent 3 μm.

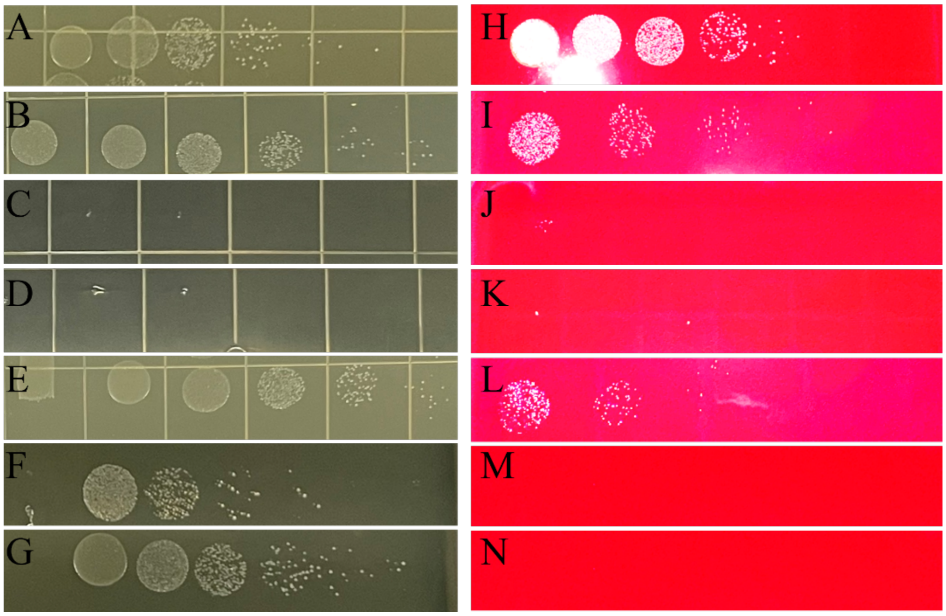

Figure S11. Representative plate images of *A. actinomycetemcomitans* (left) and *P. gingivalis* (right) colony forming units when treated for 15 min. Plates show A & H) control, B & I) 1° RSNO-MSN, C & J) 405 nm 1° RSNO-MSN, D & K) 455 nm 1° RSNO-MSN, E & L) 3° RSNO-MSN, F & M) 405 nm 3° RSNO-MSN, and G & N) 455 nm 3° RSNO-MSNs. Irradiances were 1000 mW cm^{-2}.
